# Supplementary material for: Cell-free DNA in spent culture medium effectively reflects the chromosomal status of embryos following culturing beyond implantation compared to trophectoderm biopsy
Source: PLoS One. 2021 Feb 11;16(2):e0246438. doi: 10.1371/journal.pone.0246438 (PMC7877764; doi:10.1371/journal.pone.0246438)

Embryo No1

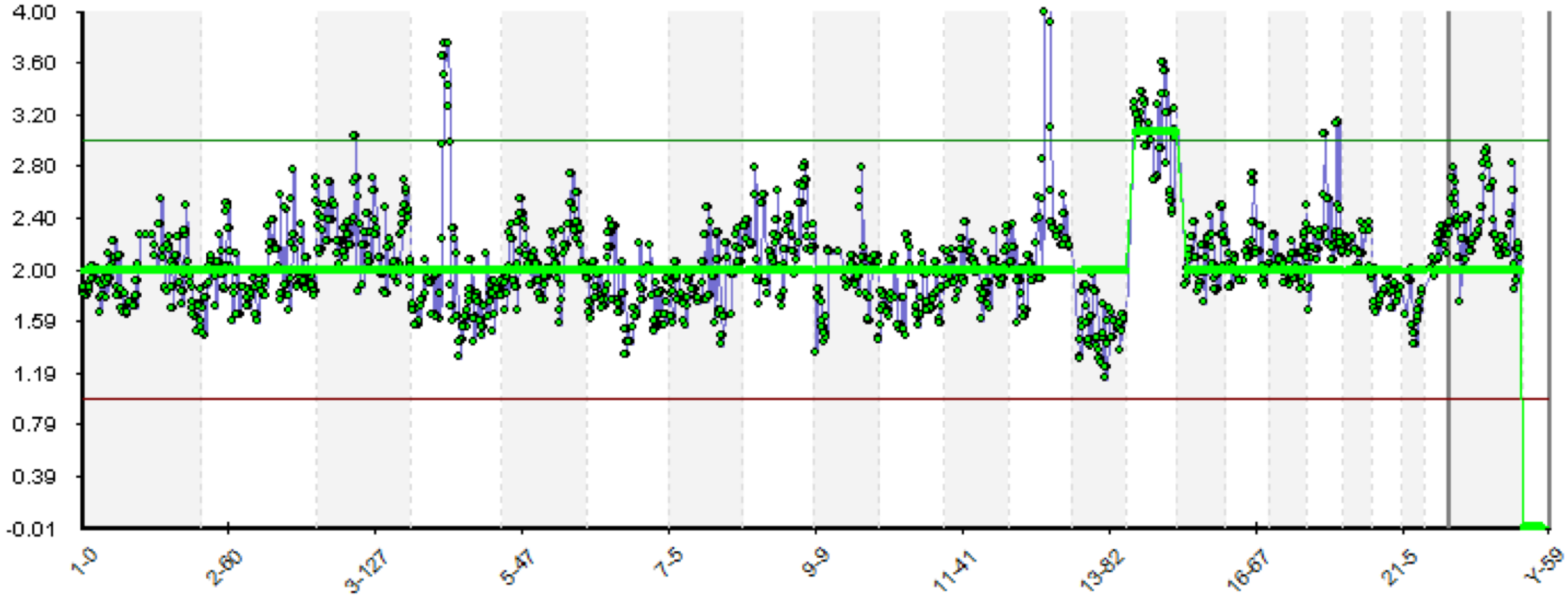

PGT-A

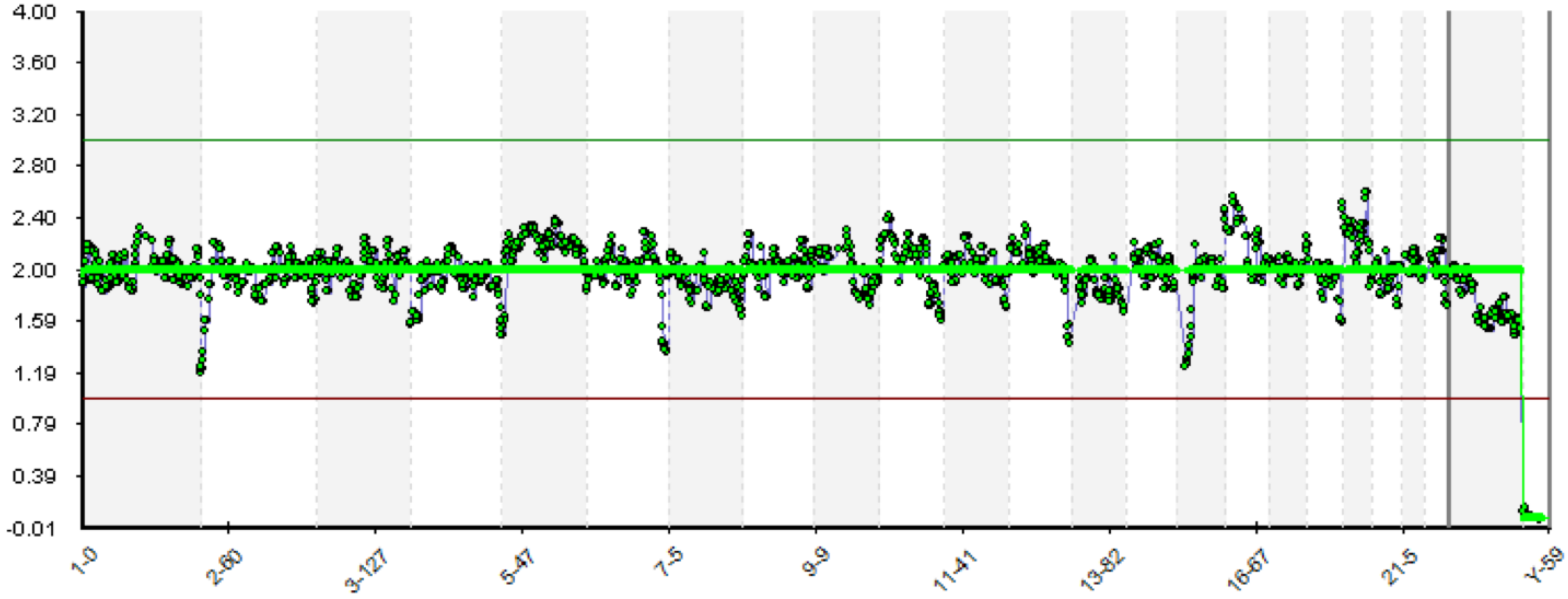

Outgrowth

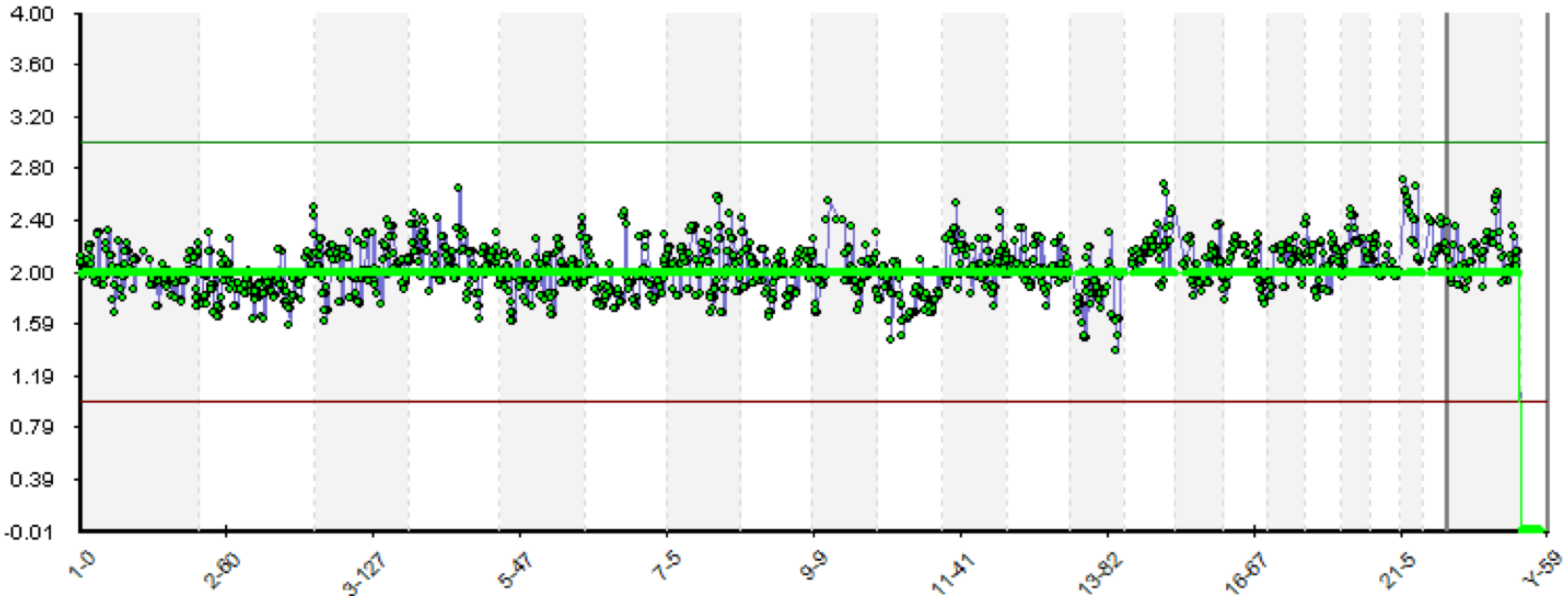

niPGT-A

Embryo No2

PGT-A

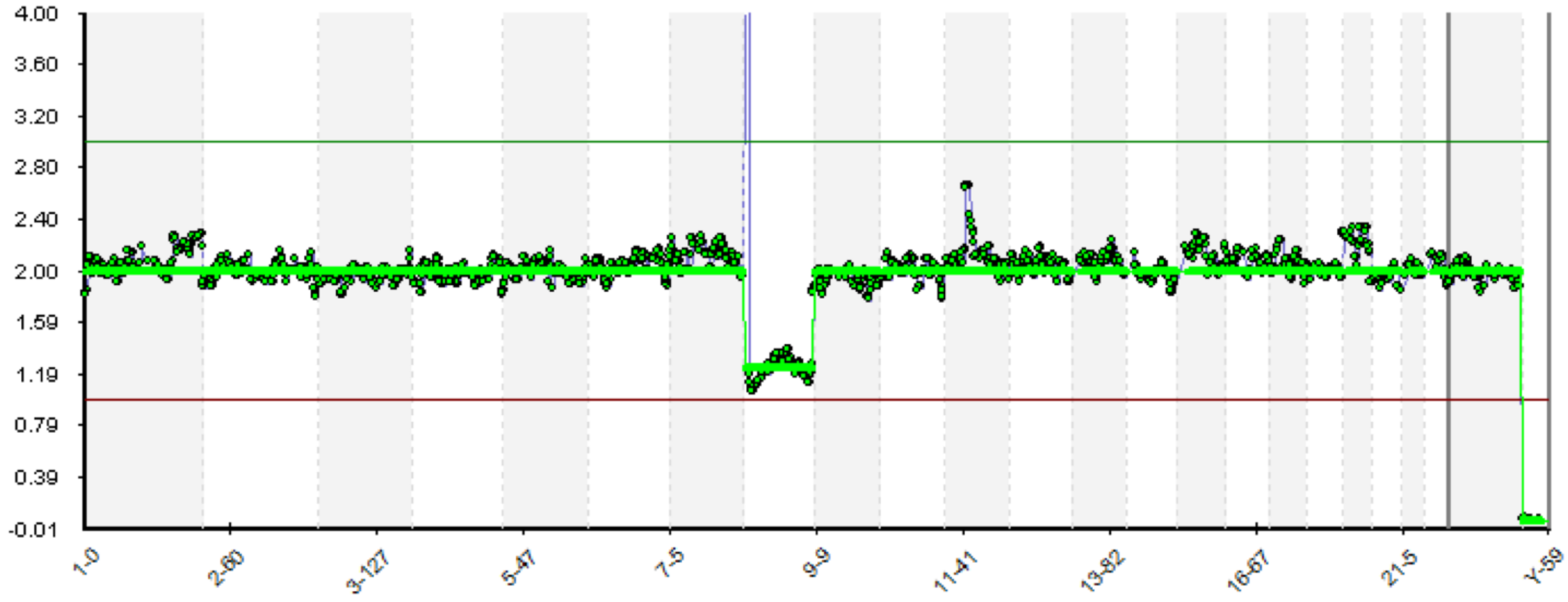

Outgrowth

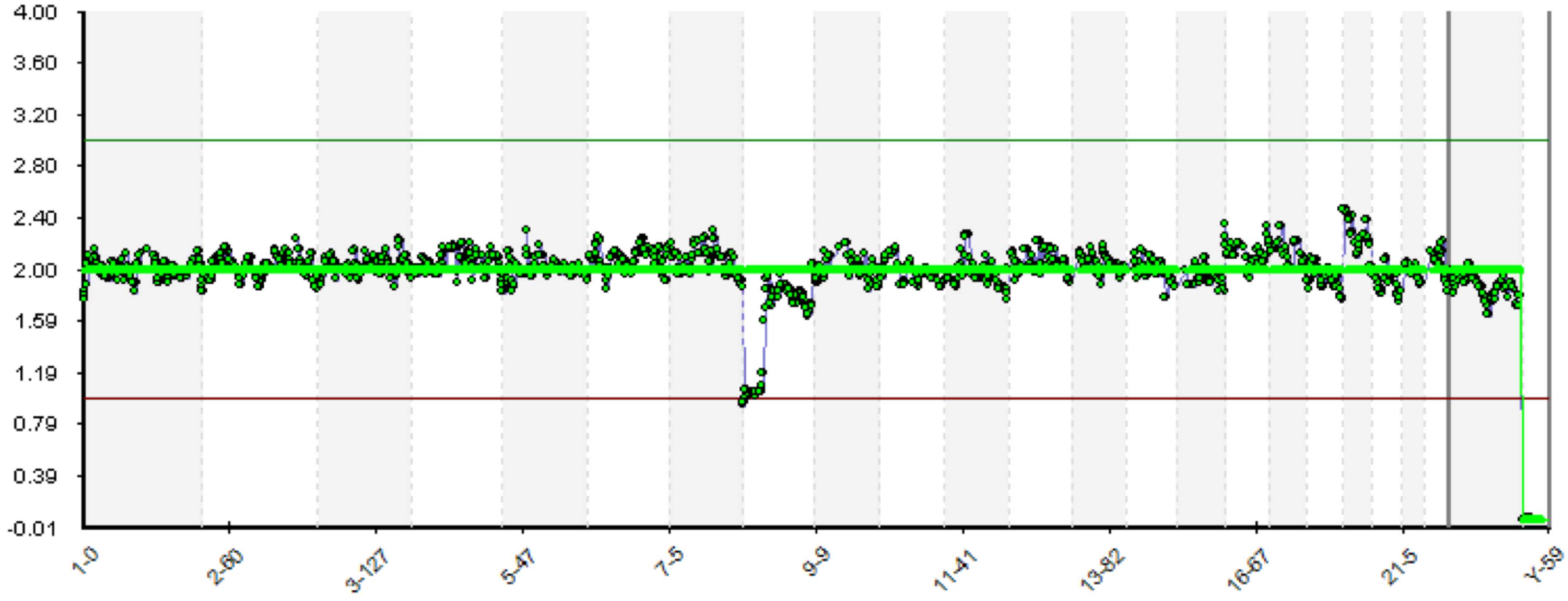

niPGT-A

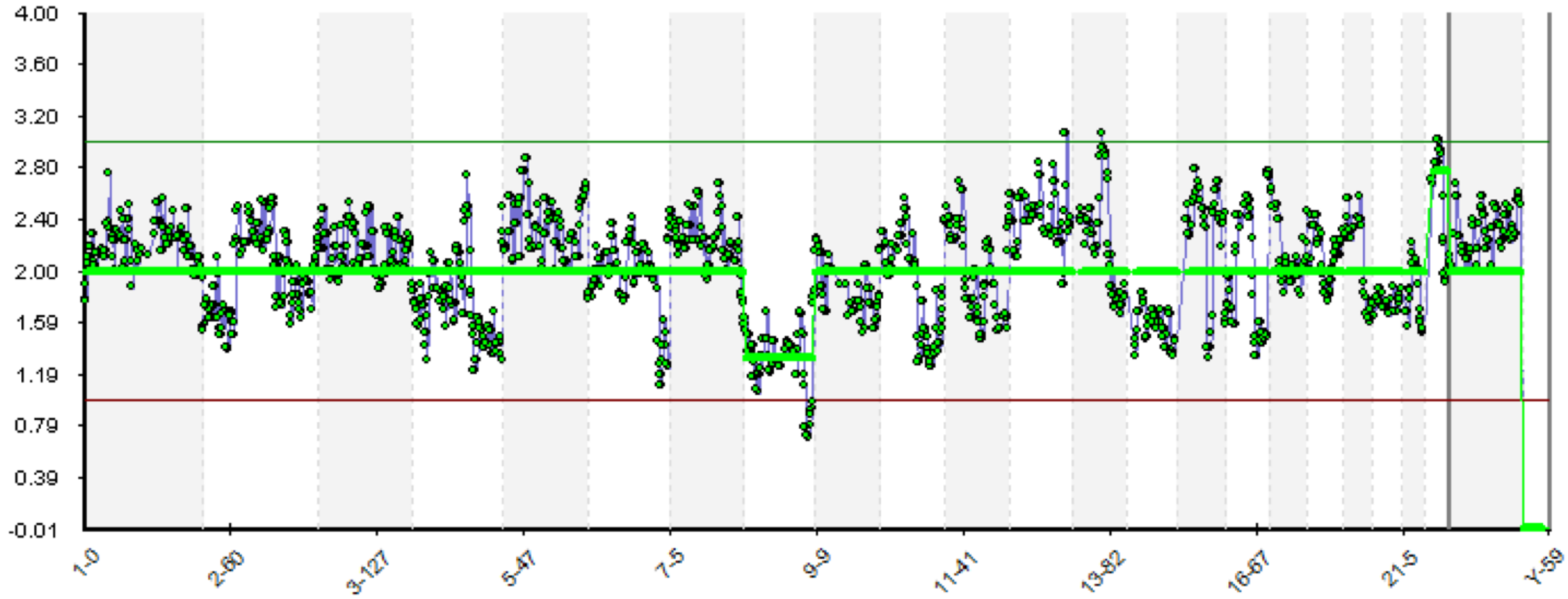

Embryo No3

PGT-A

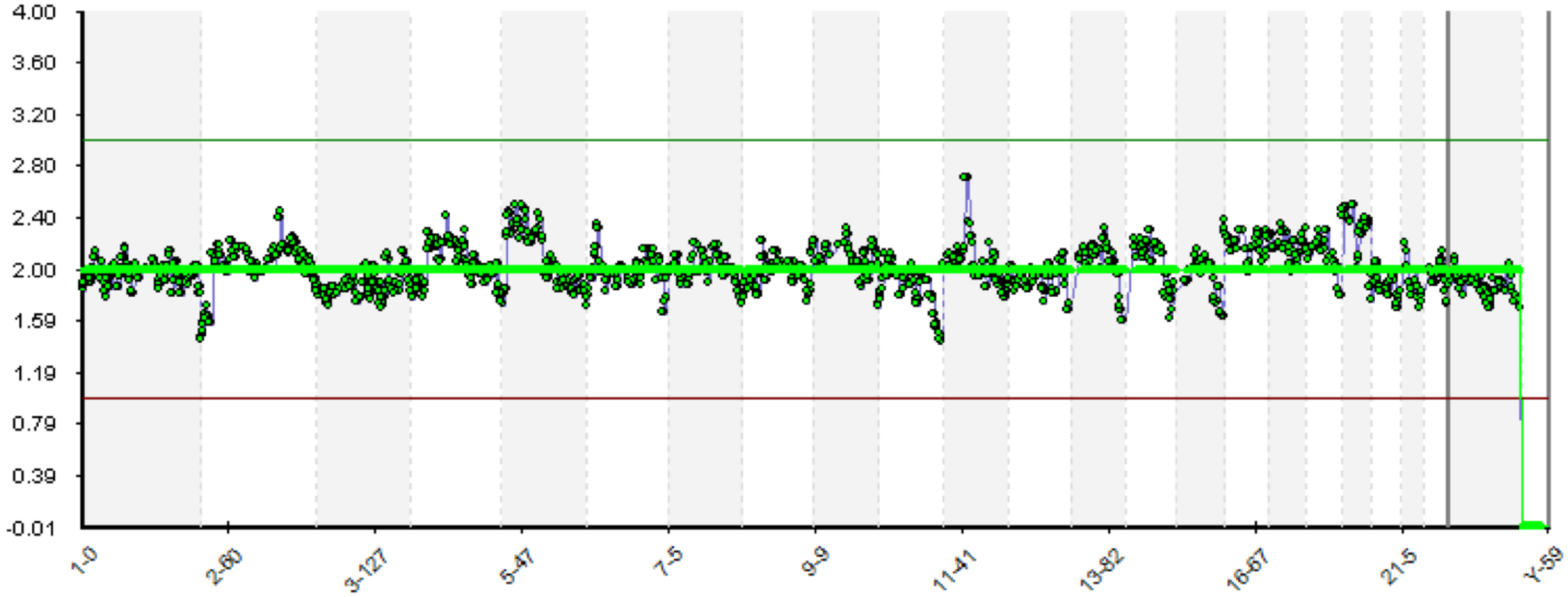

Outgrowth

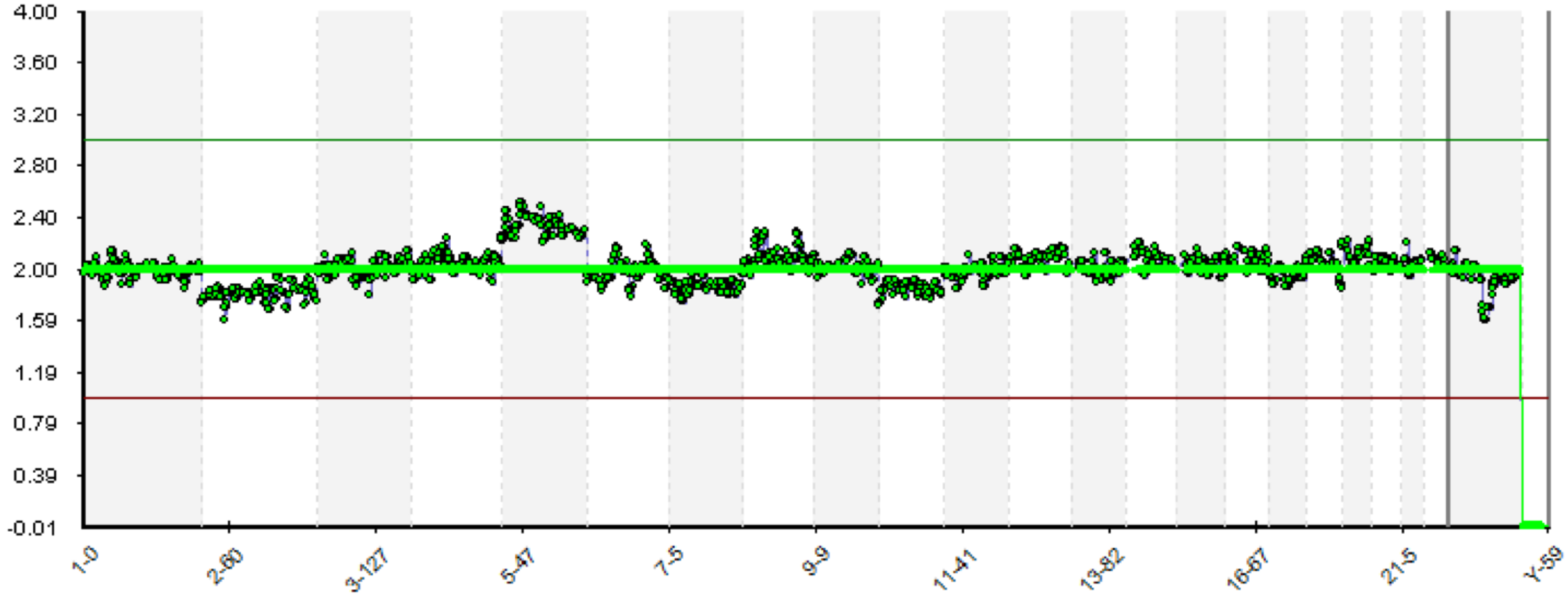

niPGT-A

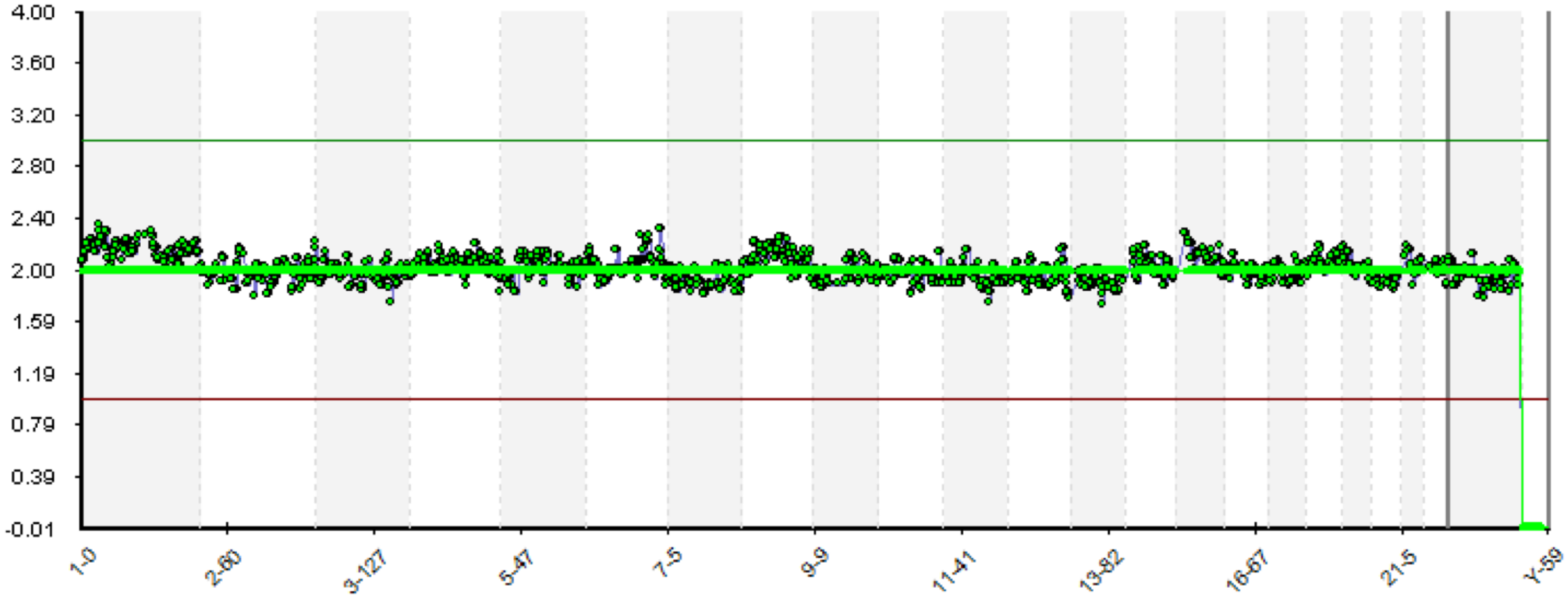

Embryo No4

PGT-A

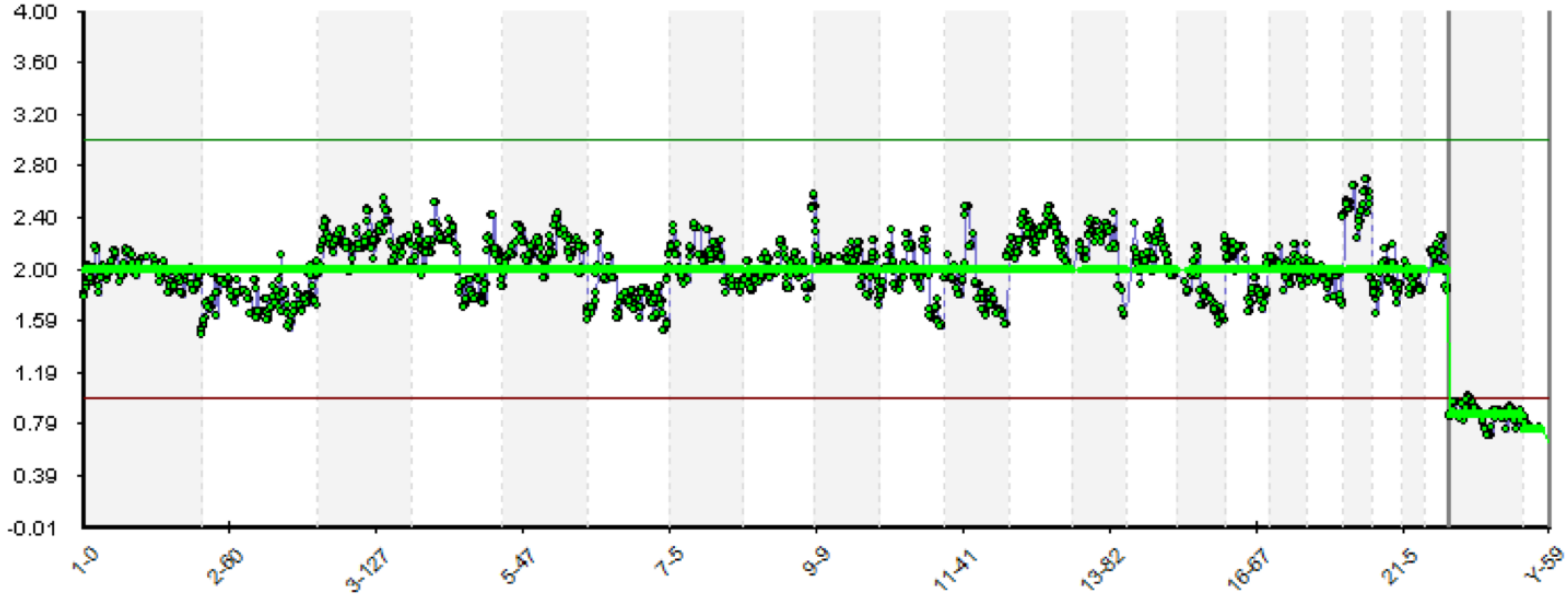

Outgrowth

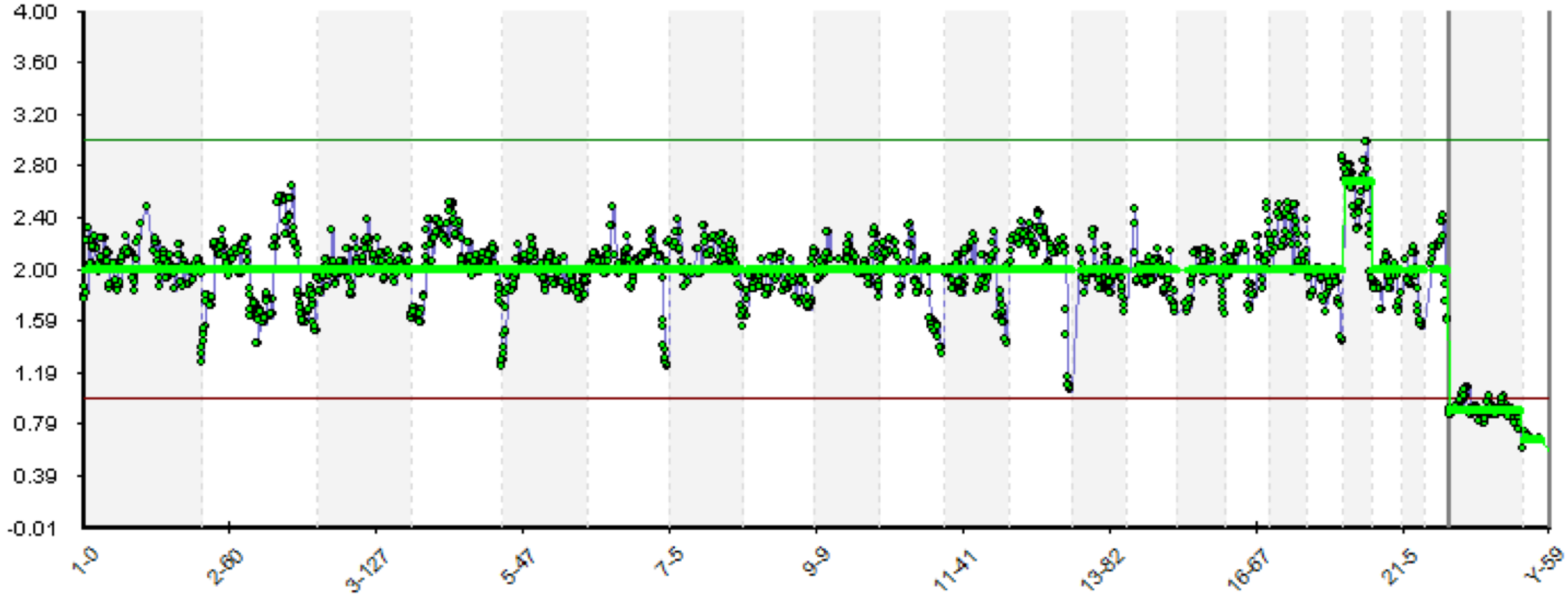

niPGT-A

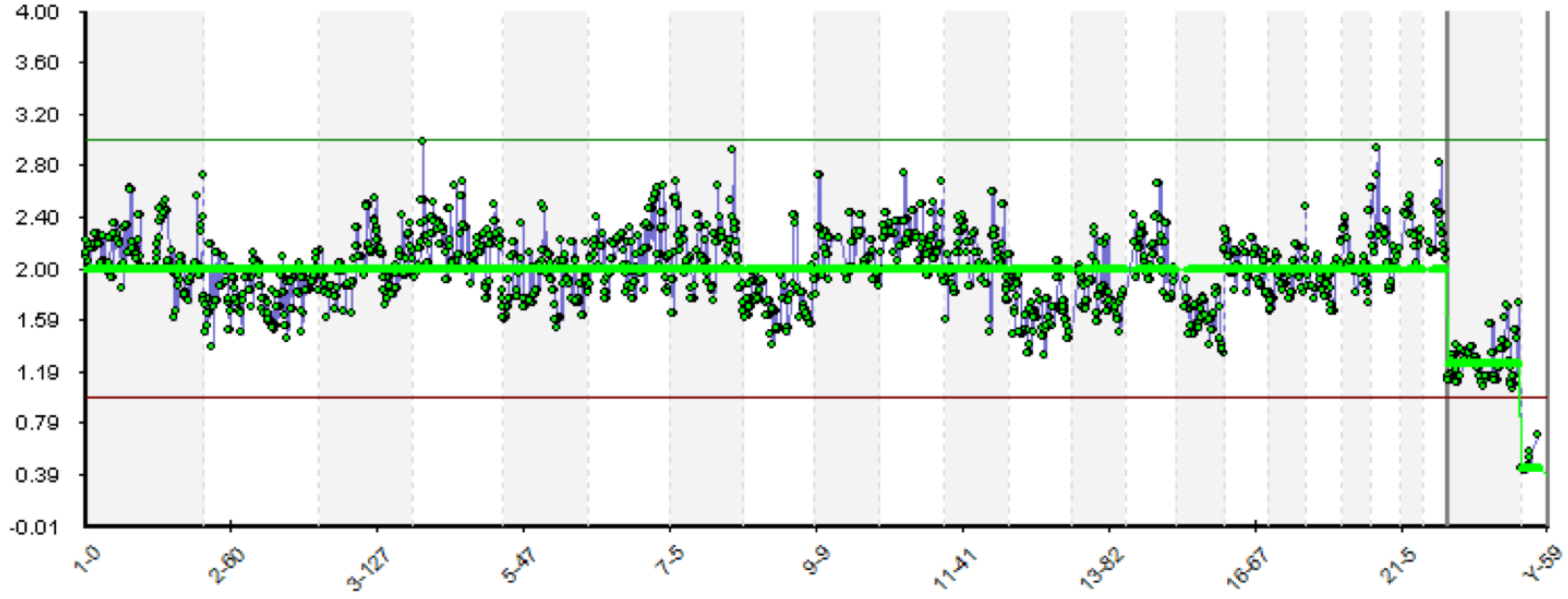

Embryo No5

PGT-A

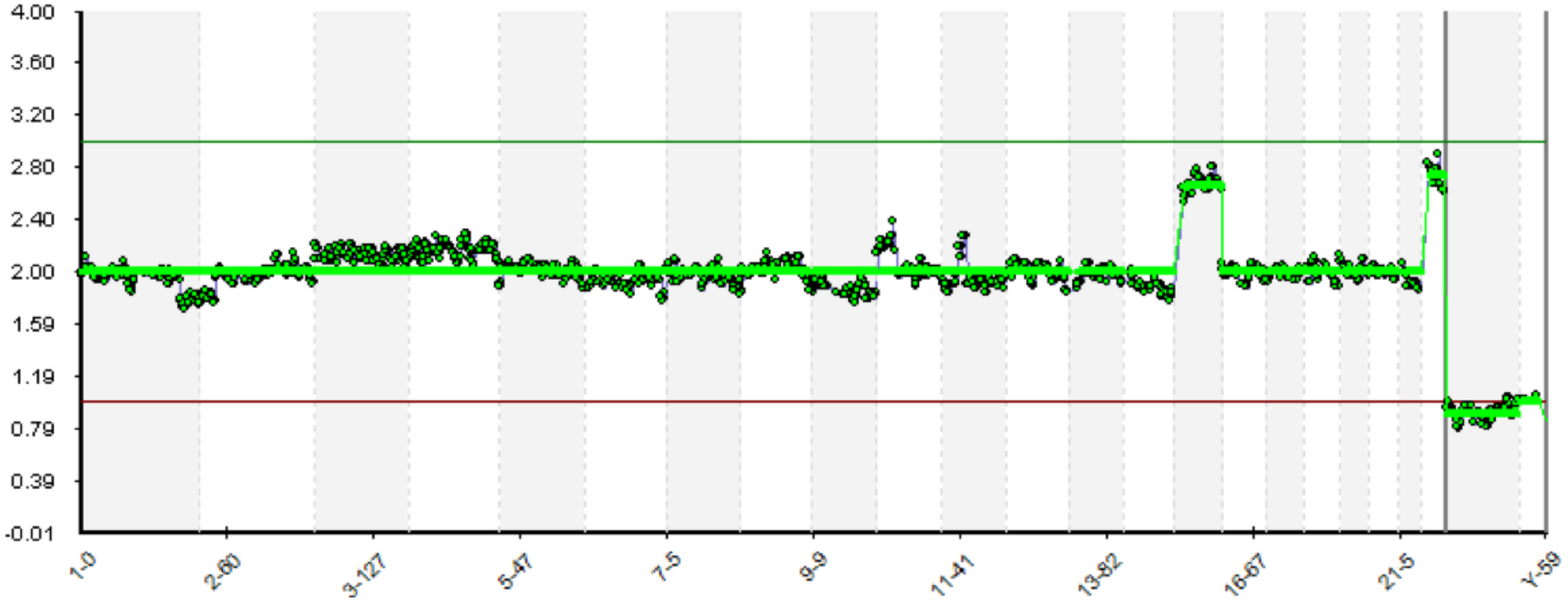

Outgrowth

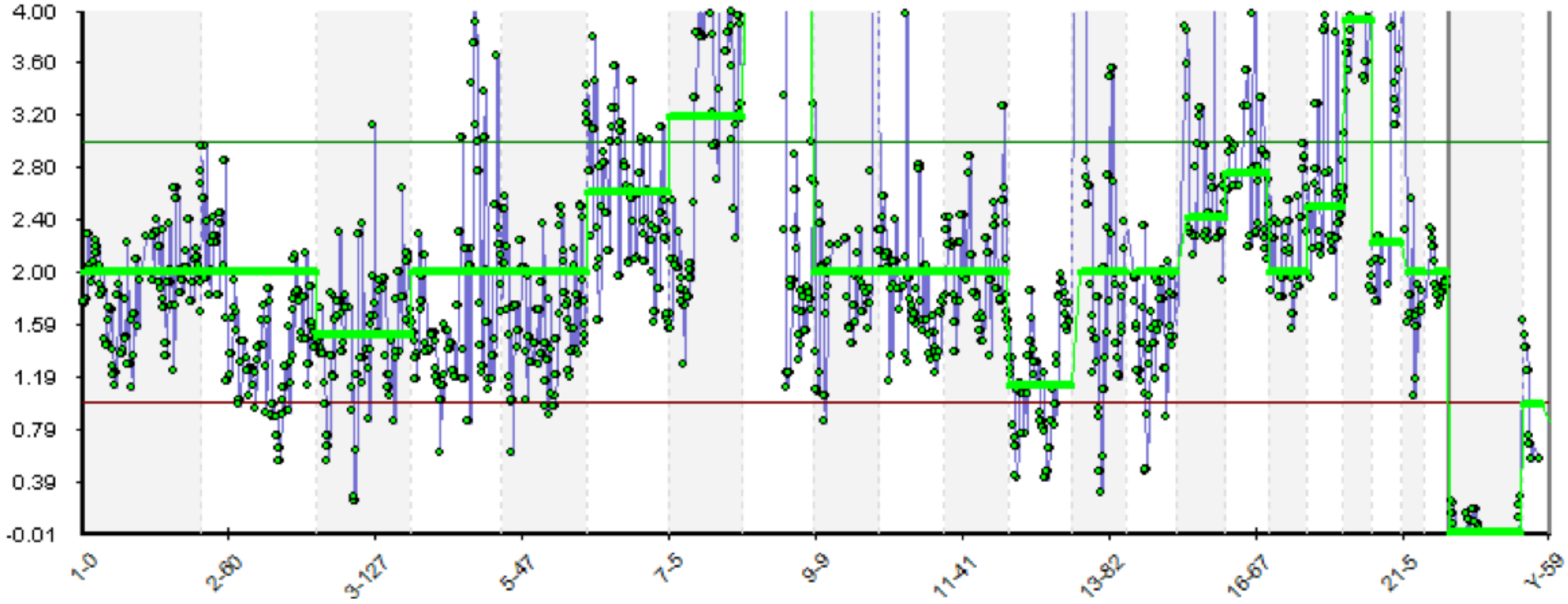

niPGT-A

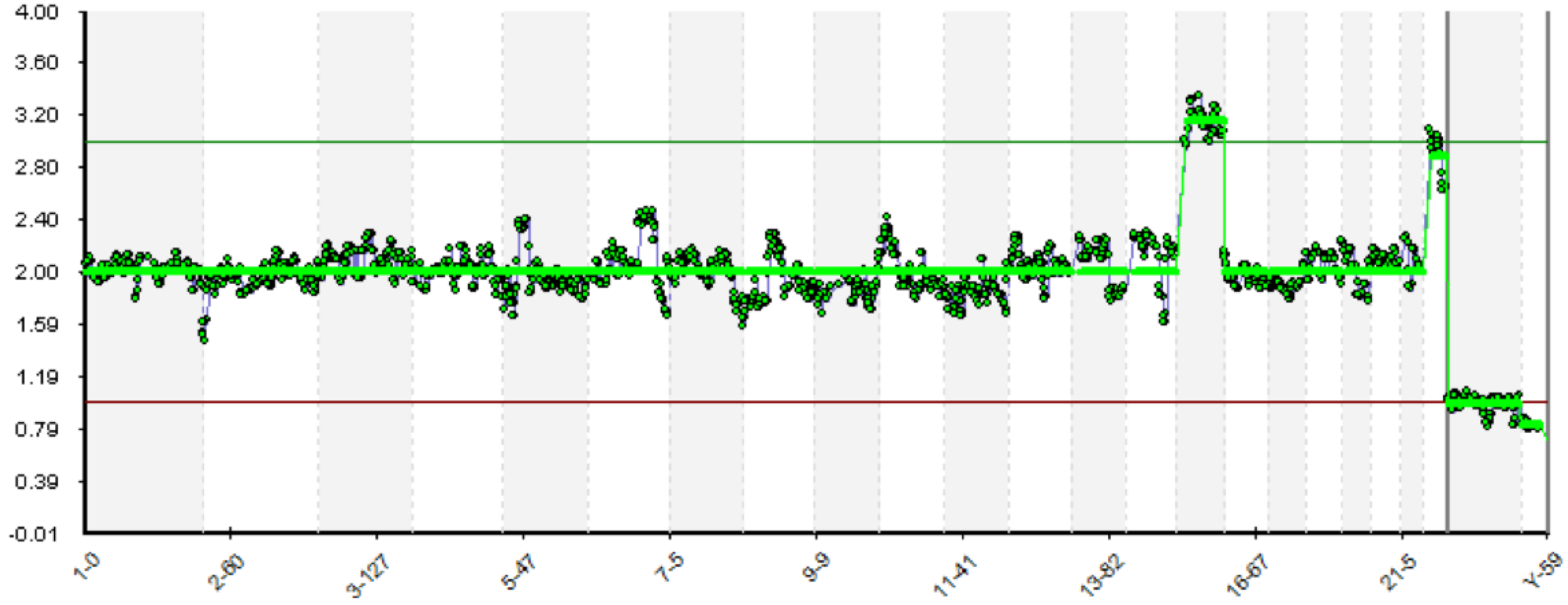

Embryo No6

PGT-A

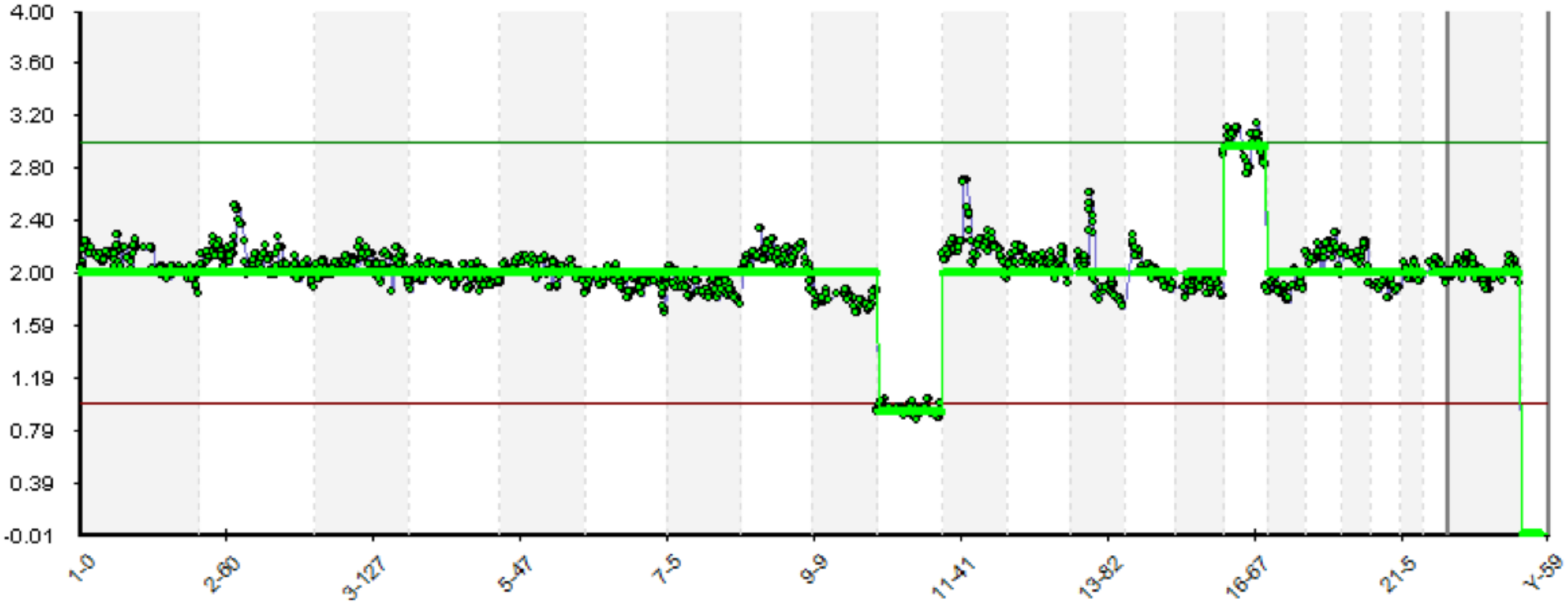

Outgrowth

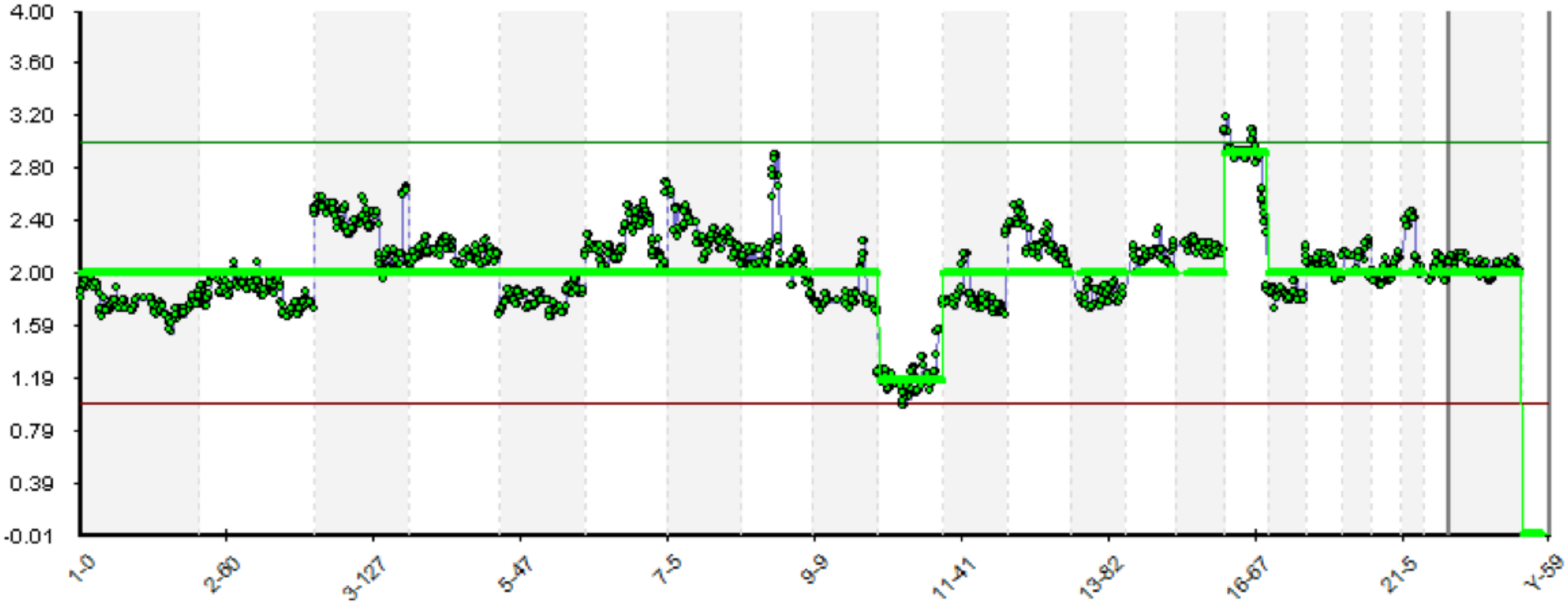

niPGT-A

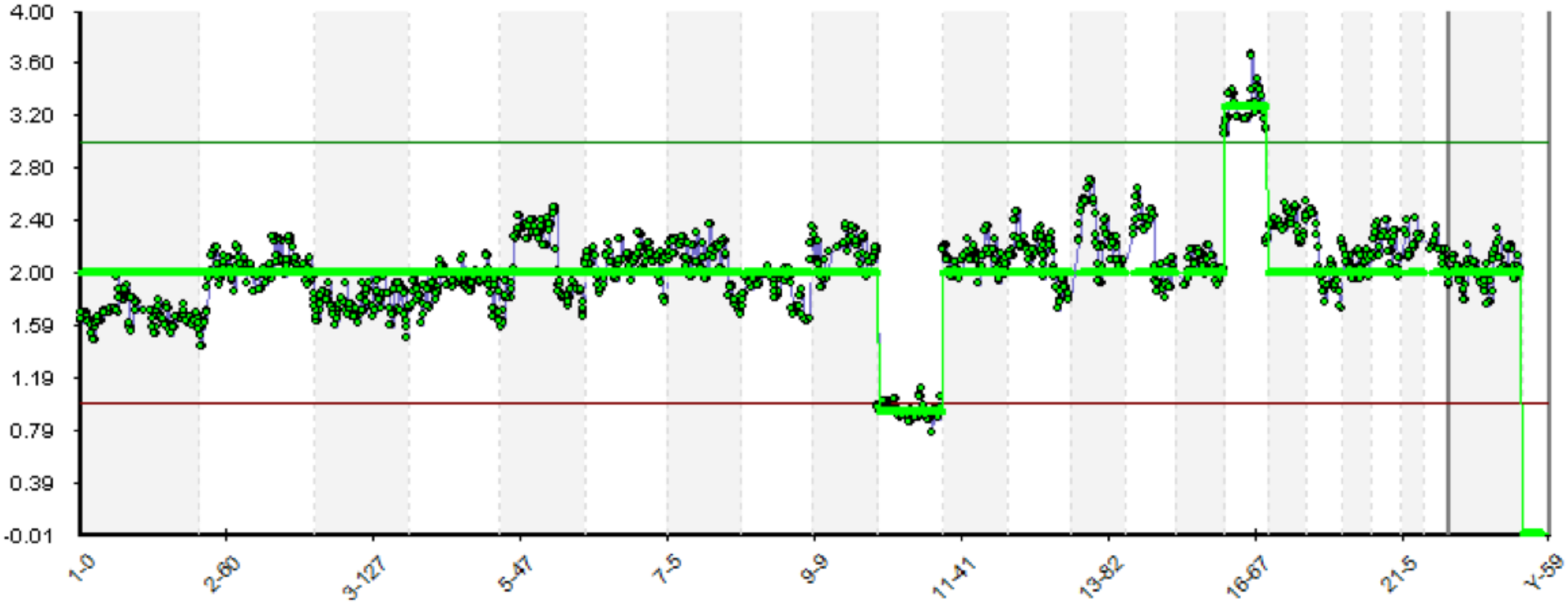

Embryo No7

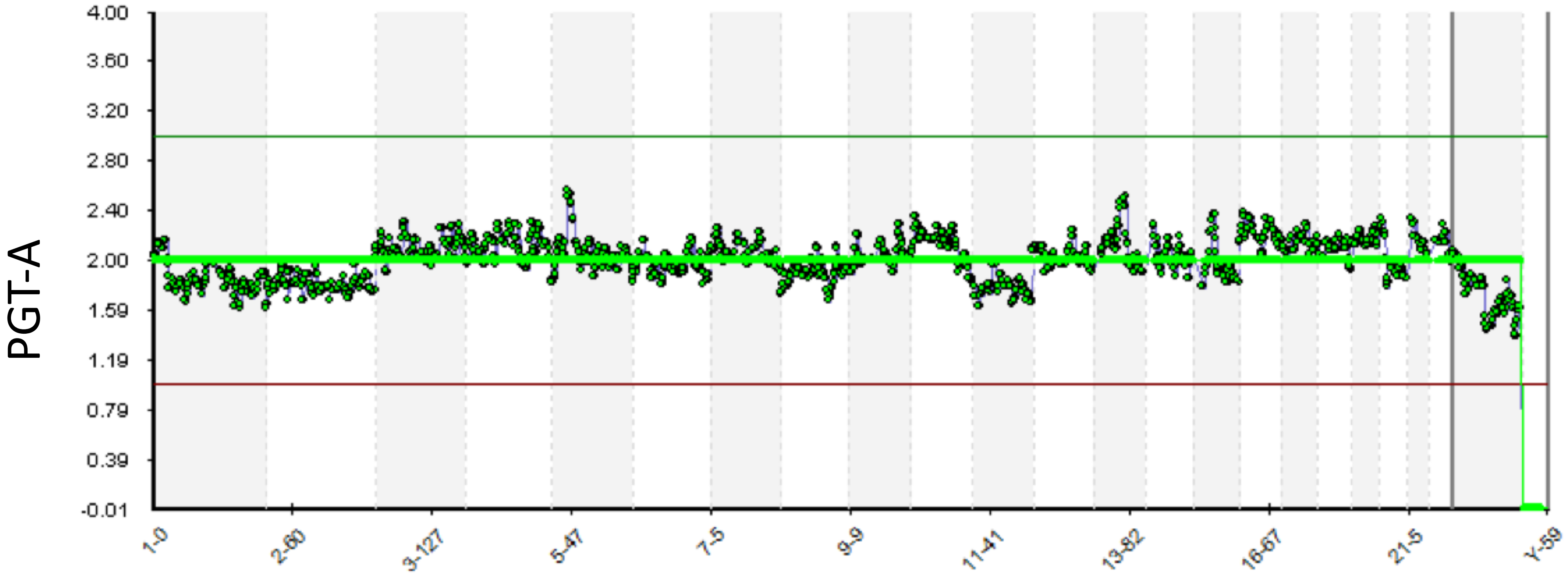
PGT-A
4.00
3.60
3.20
2.80
2.40
2.00
1.59
1.19
0.79
0.39
-0.01
1-0
2-60
3-127
5-47
7-5
9-9
11-41
13-82
16-67
21-5
Y-59

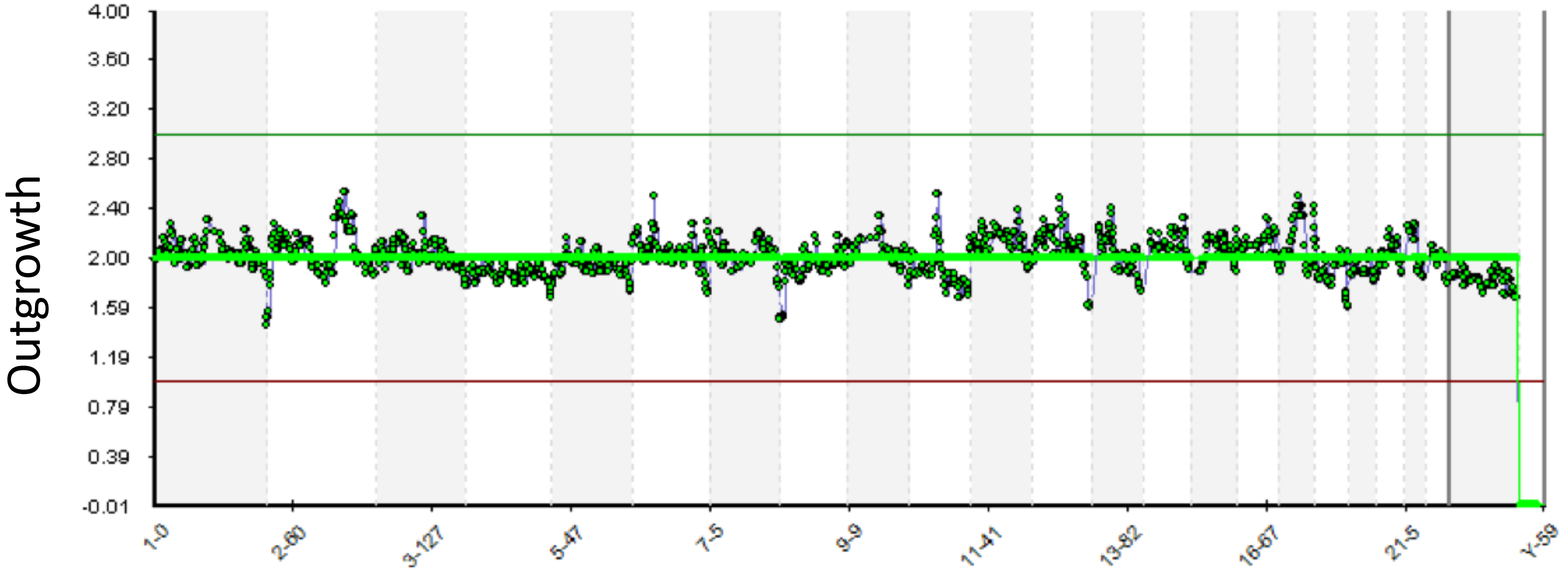
Outgrowth
4.00
3.60
3.20
2.80
2.40
2.00
1.59
1.19
0.79
0.39
-0.01
1-0
2-60
3-127
5-47
7-5
9-9
11-41
13-82
16-67
21-5
Y-59

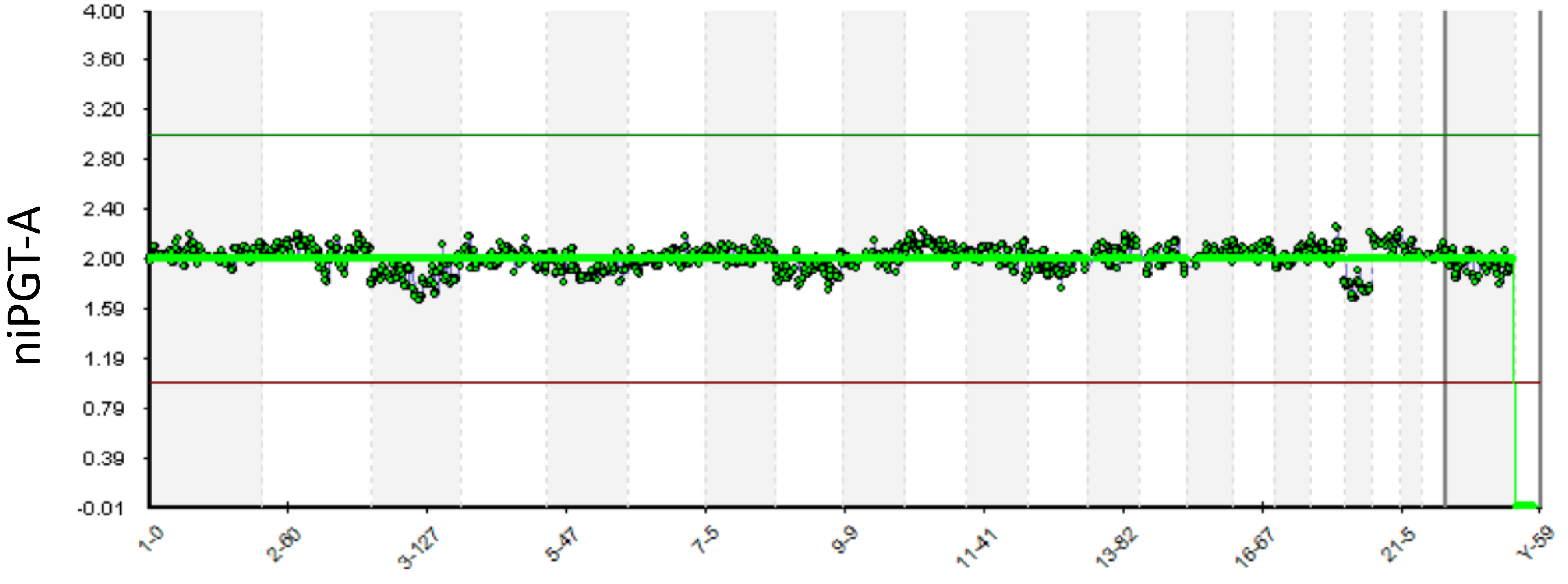
niPGT-A
4.00
3.60
3.20
2.80
2.40
2.00
1.59
1.19
0.79
0.39
-0.01
1-0
2-60
3-127
5-47
7-5
9-9
11-41
13-82
16-67
21-5
Y-59

Embryo No8

PGT-A

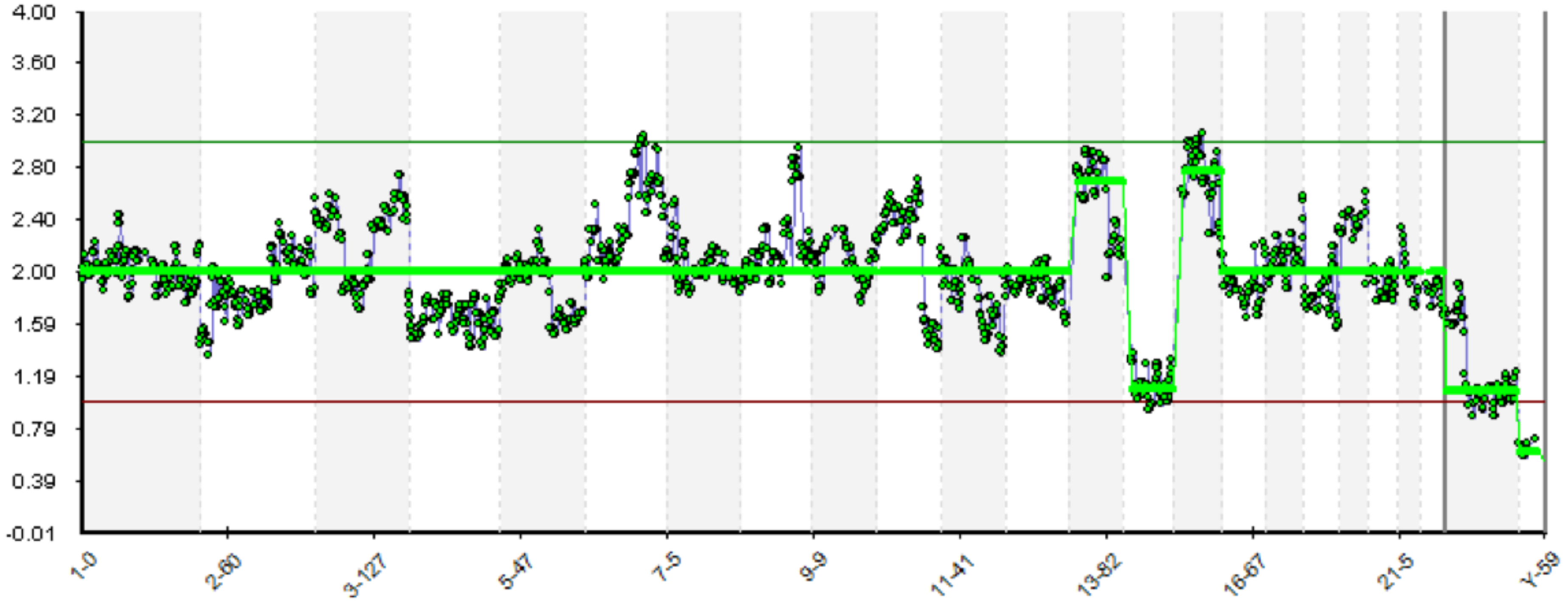

Outgrowth

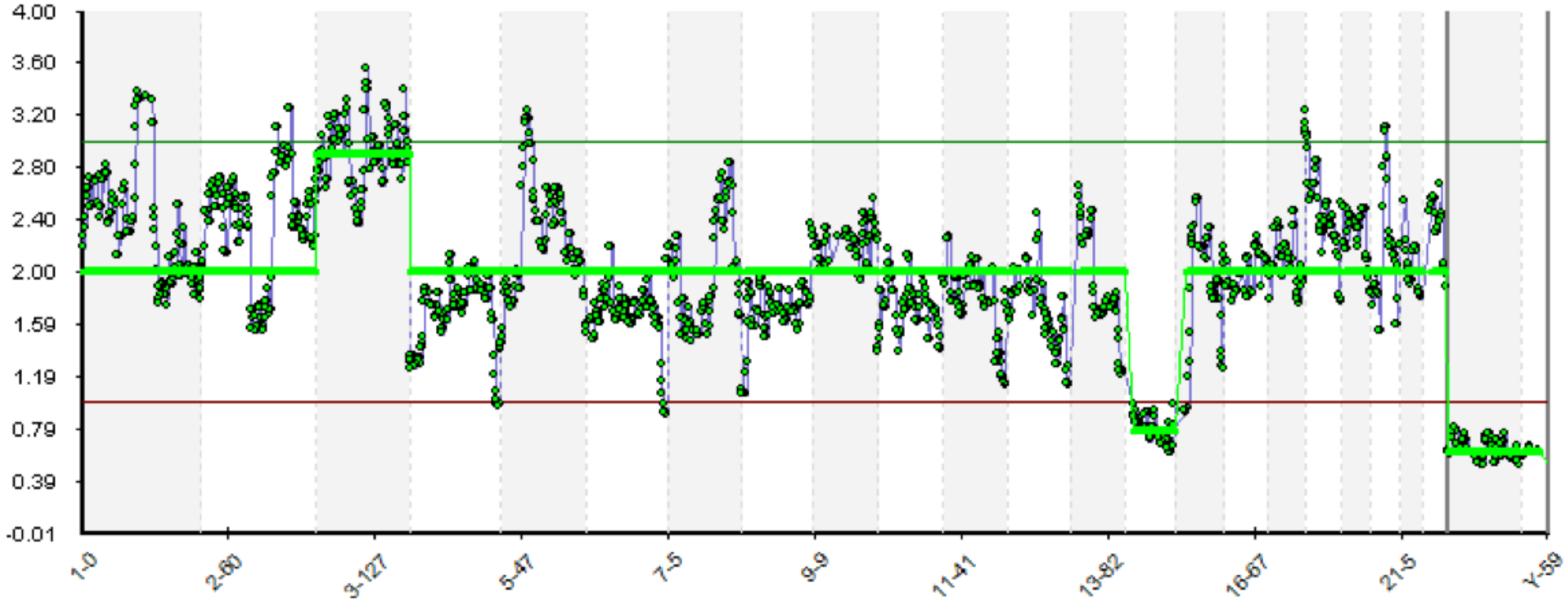

niPGT-A

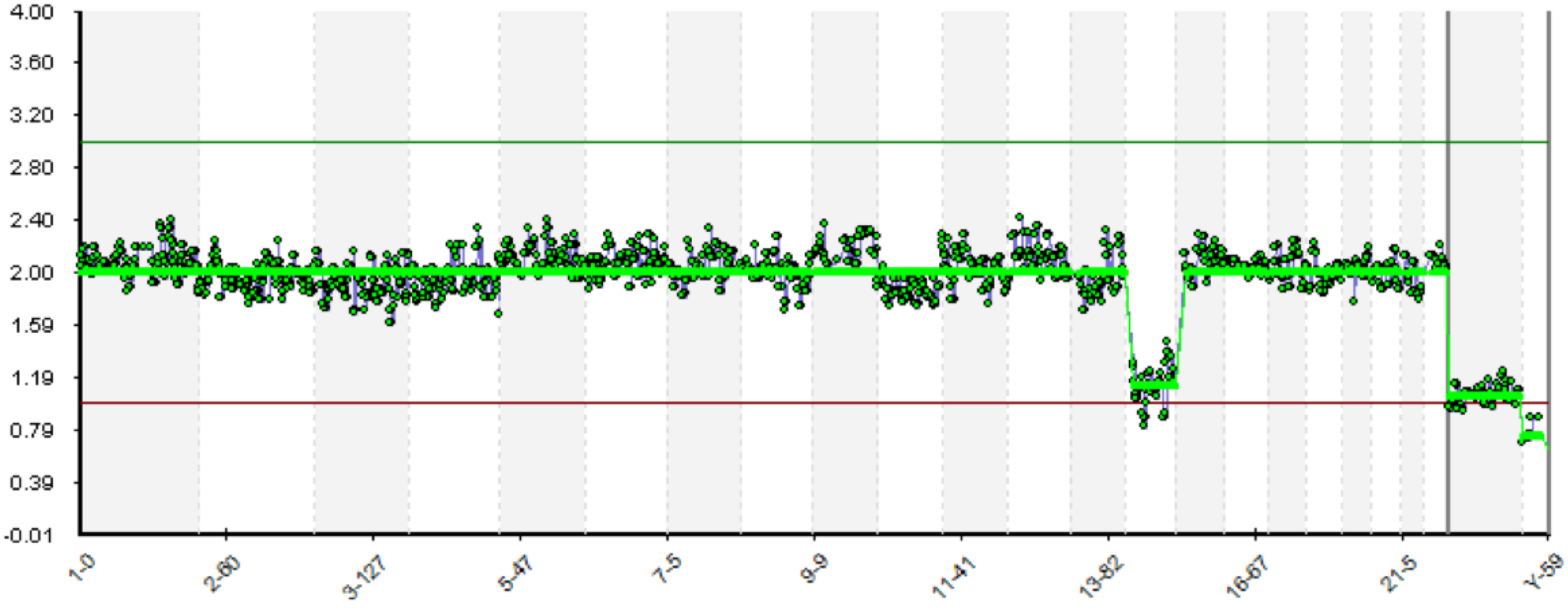

Embryo No9

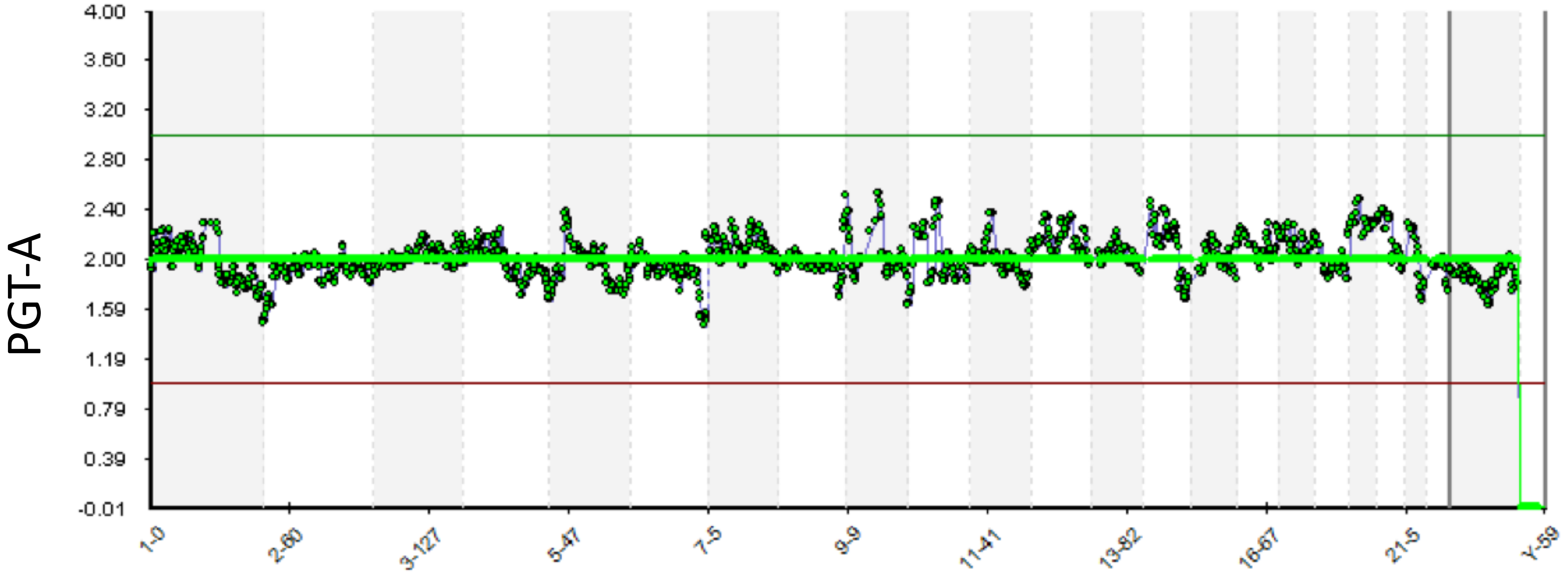
PGT-A
4.00
3.60
3.20
2.80
2.40
2.00
1.59
1.19
0.79
0.39
-0.01
1-0
2-60
3-127
5-47
7-5
9-9
11-41
13-82
16-67
21-5
Y-59

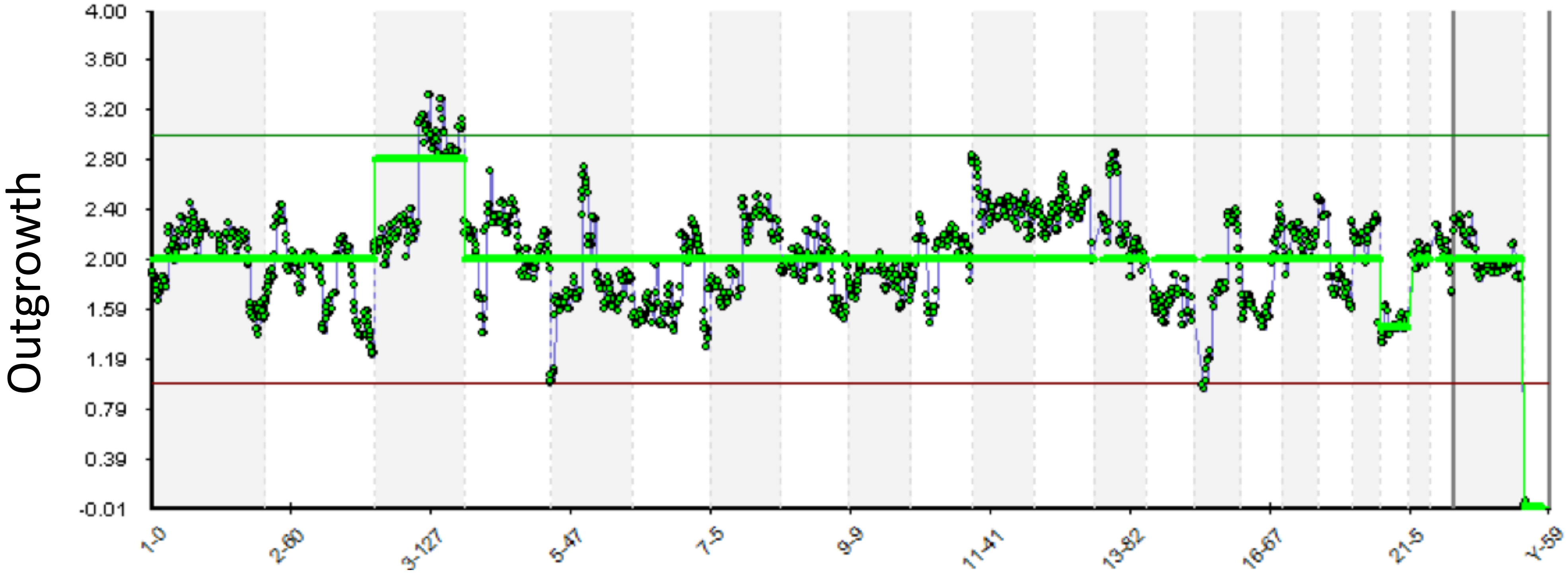
Outgrowth
4.00
3.60
3.20
2.80
2.40
2.00
1.59
1.19
0.79
0.39
-0.01
1-0
2-60
3-127
5-47
7-5
9-9
11-41
13-82
16-67
21-5
Y-59

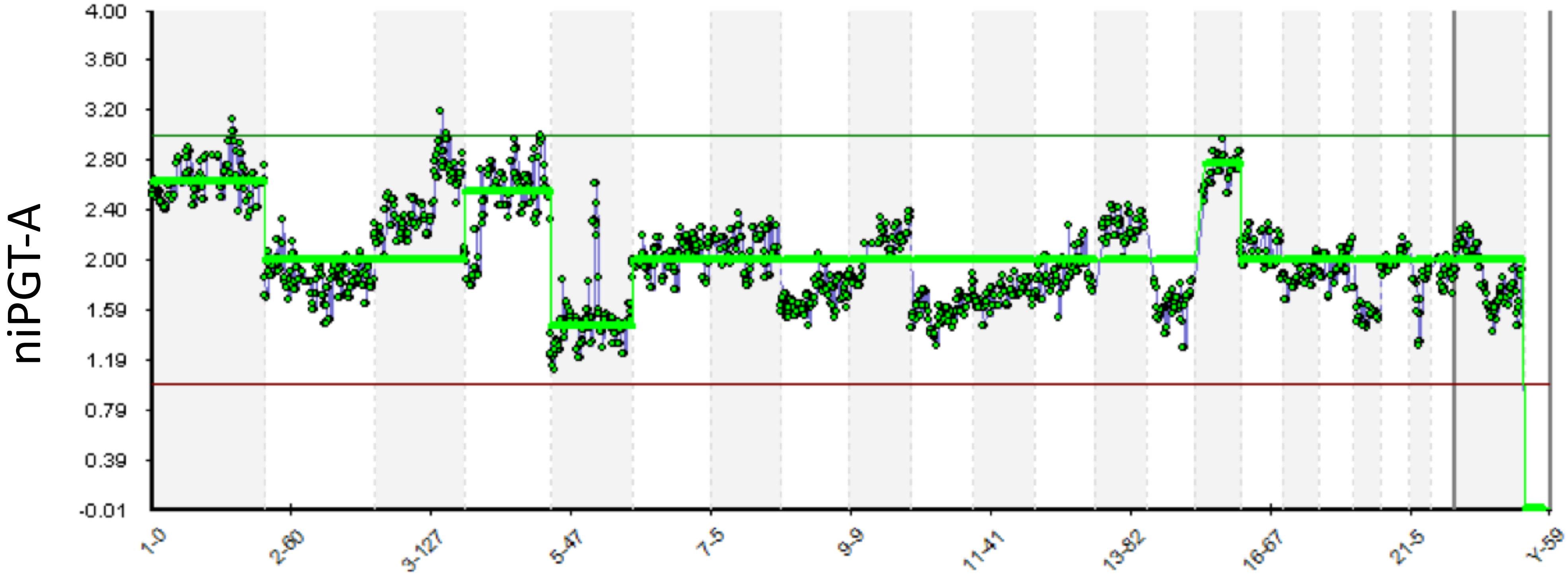
niPGT-A
4.00
3.60
3.20
2.80
2.40
2.00
1.59
1.19
0.79
0.39
-0.01
1-0
2-60
3-127
5-47
7-5
9-9
11-41
13-82
16-67
21-5
Y-59

Embryo No10

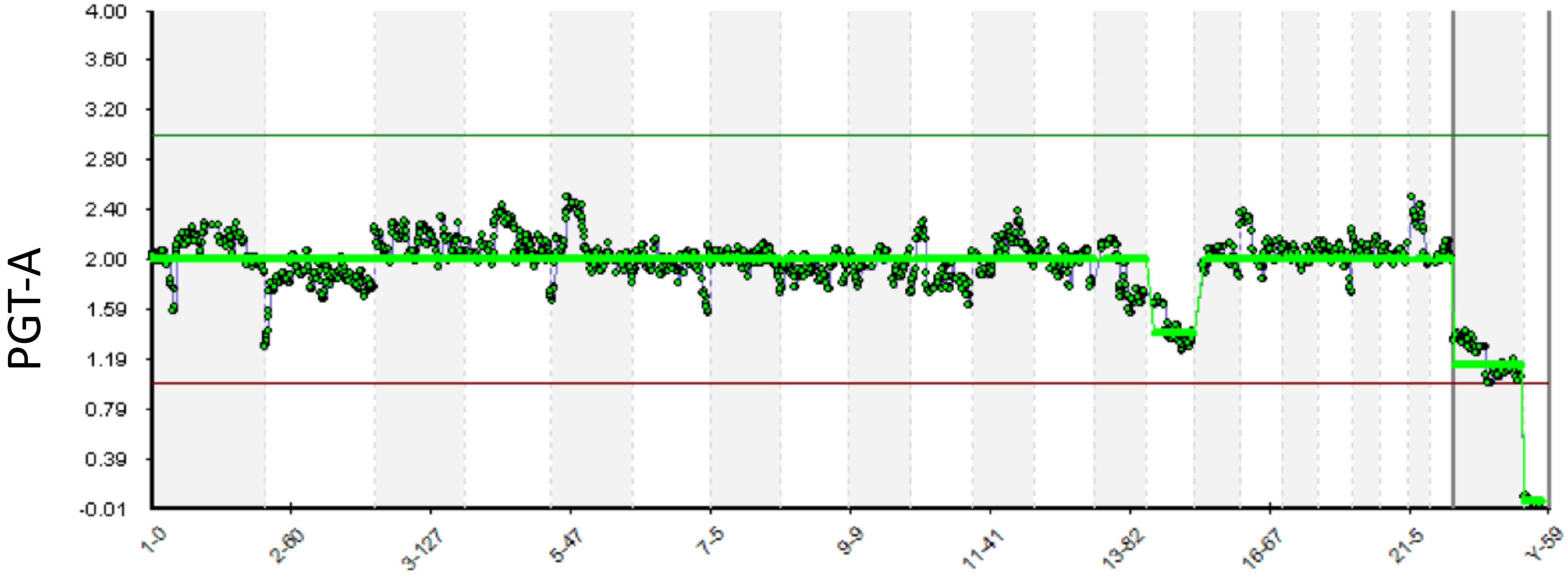
PGT-A
4.00
3.60
3.20
2.80
2.40
2.00
1.59
1.19
0.79
0.39
-0.01
1-0
2-60
3-127
5-47
7-5
9-9
11-41
13-82
16-67
21-5
Y-59

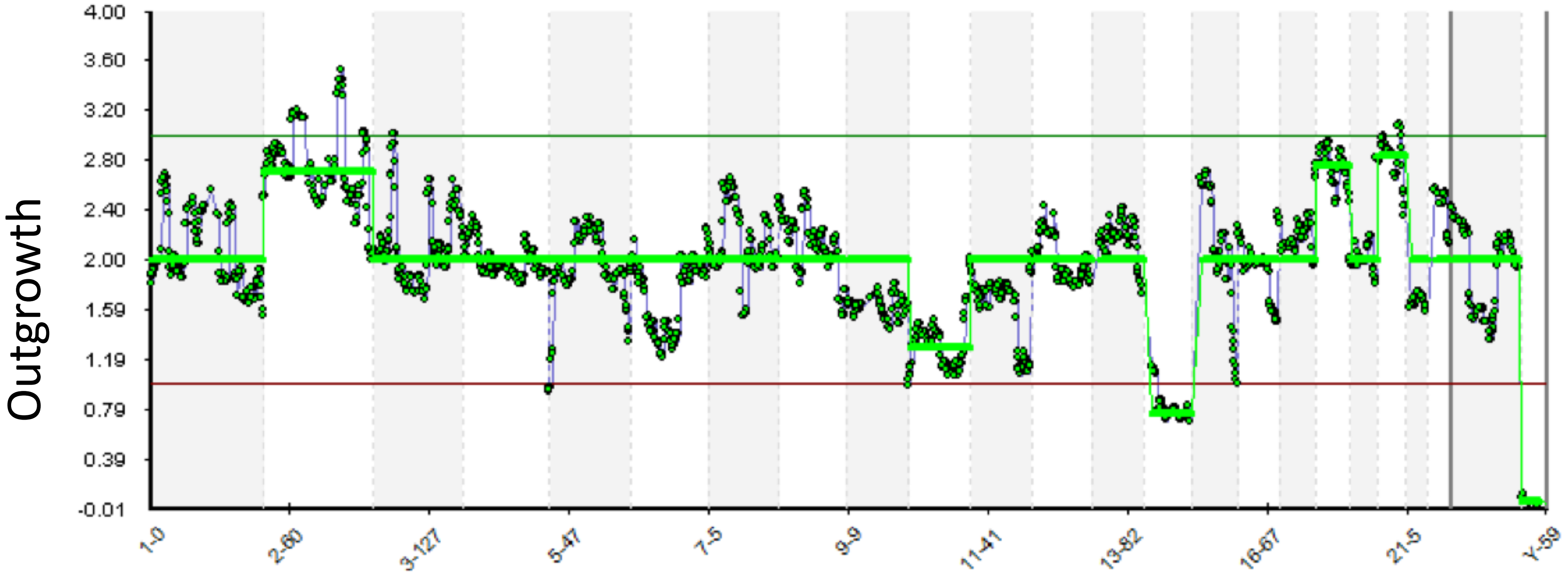
Outgrowth
4.00
3.60
3.20
2.80
2.40
2.00
1.59
1.19
0.79
0.39
-0.01
1-0
2-60
3-127
5-47
7-5
9-9
11-41
13-82
16-67
21-5
Y-59

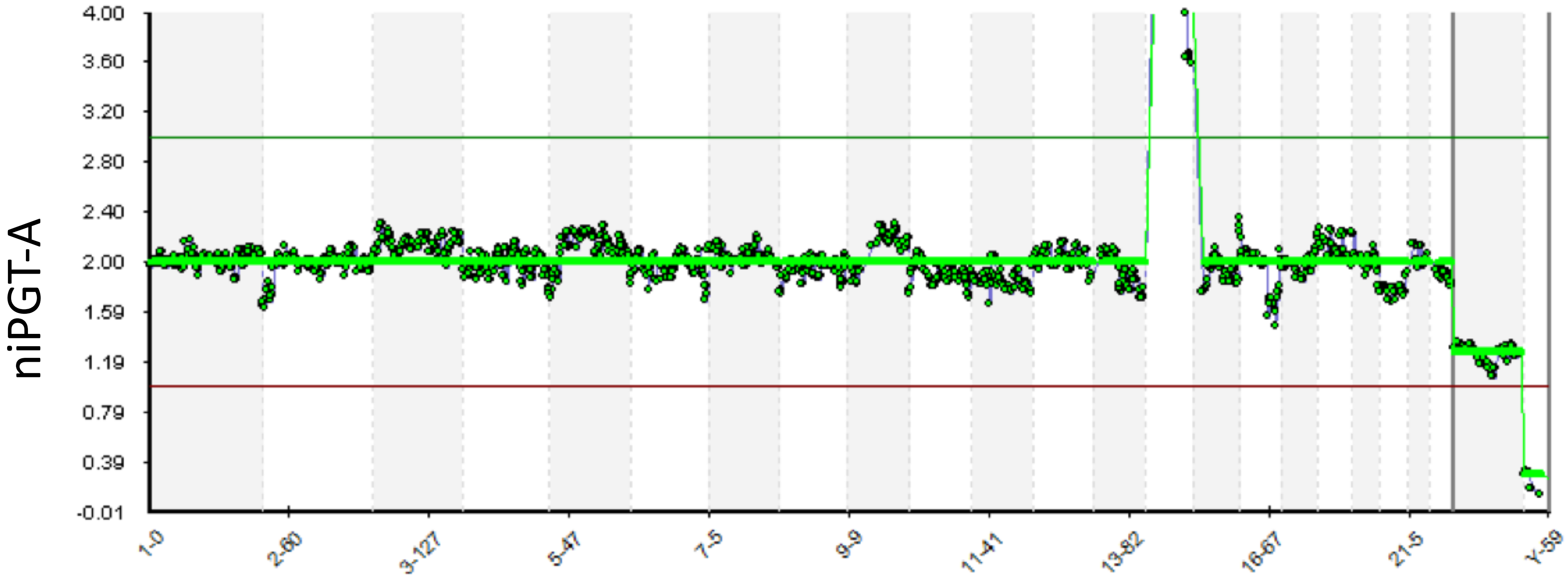
niPGT-A
4.00
3.60
3.20
2.80
2.40
2.00
1.59
1.19
0.79
0.39
-0.01
1-0
2-60
3-127
5-47
7-5
9-9
11-41
13-82
16-67
21-5
Y-59

Embryo No11

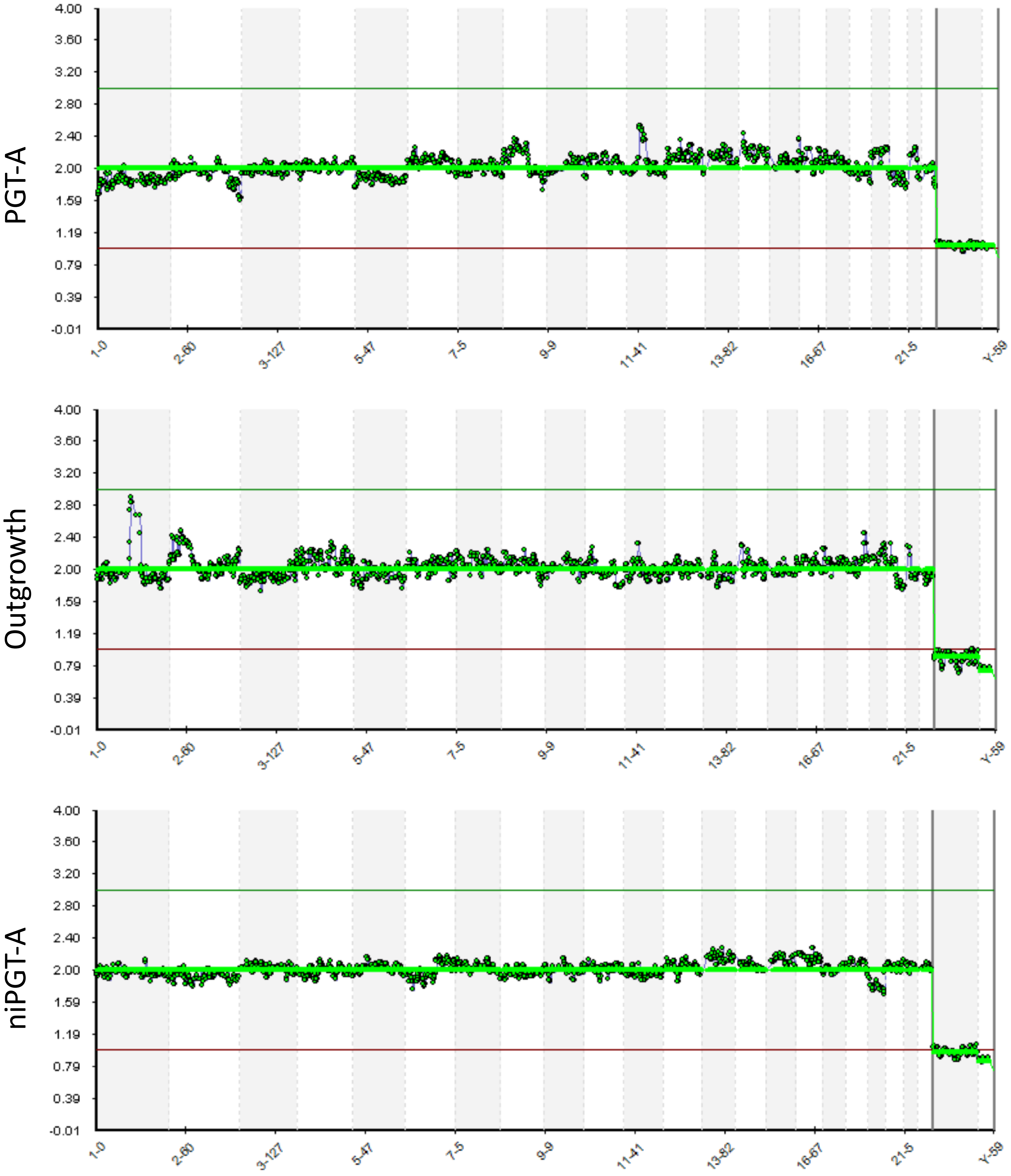

PGT-A
Outgrowth
niPGT-A
4.00
3.60
3.20
2.80
2.40
2.00
1.59
1.19
0.79
0.39
-0.01
1-0
2-60
3-127
5-47
7-5
9-9
11-41
13-82
16-67
21-5
Y-59

Embryo No6

PGT-A

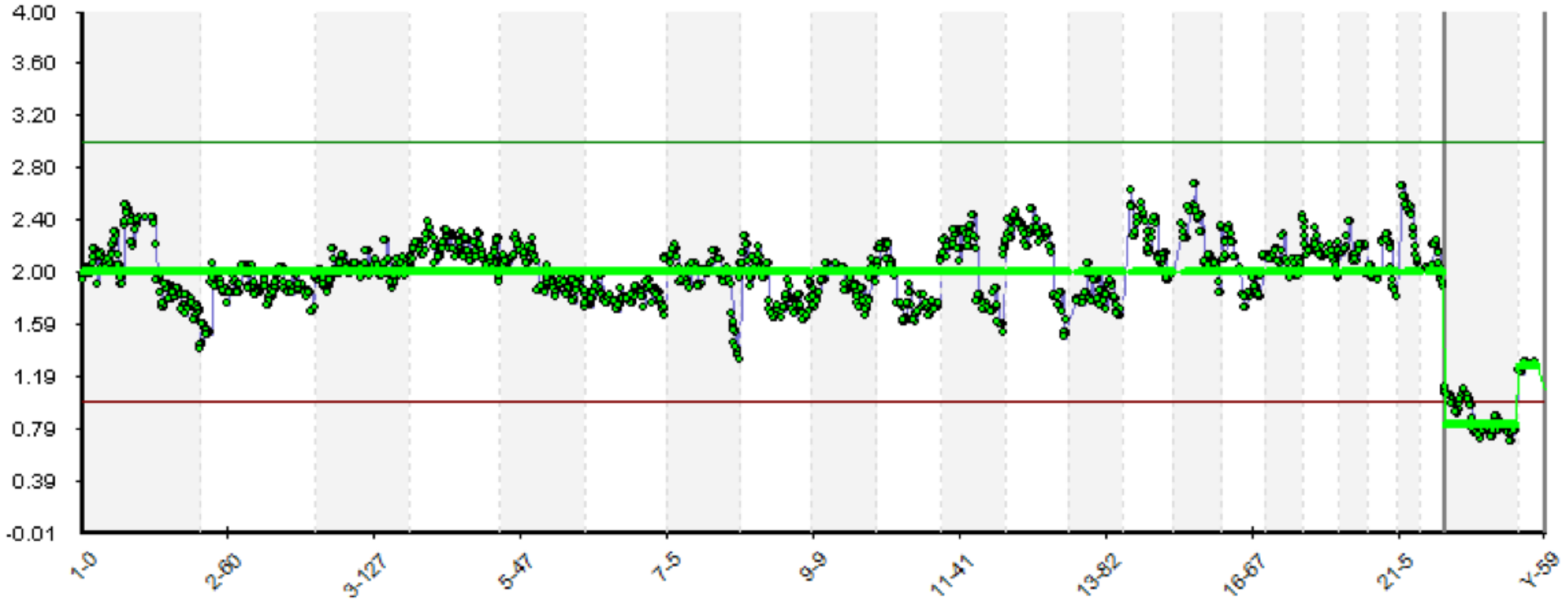

Outgrowth

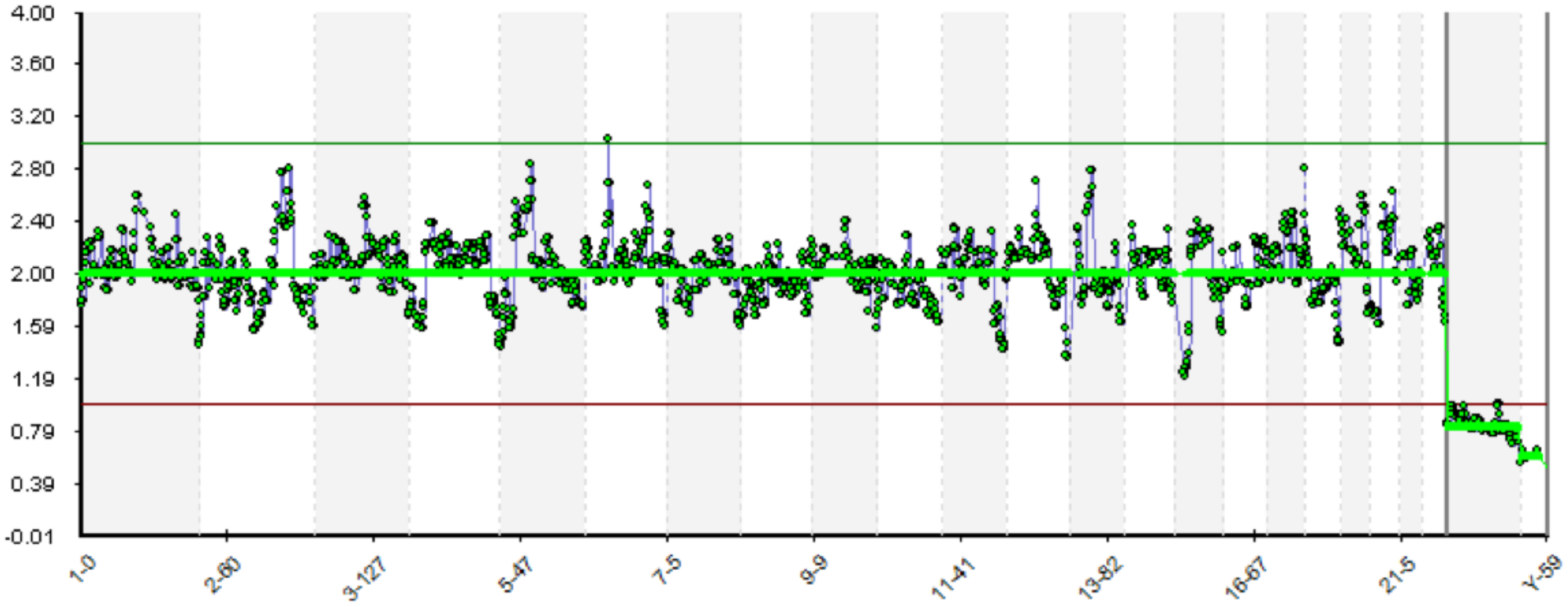

niPGT-A

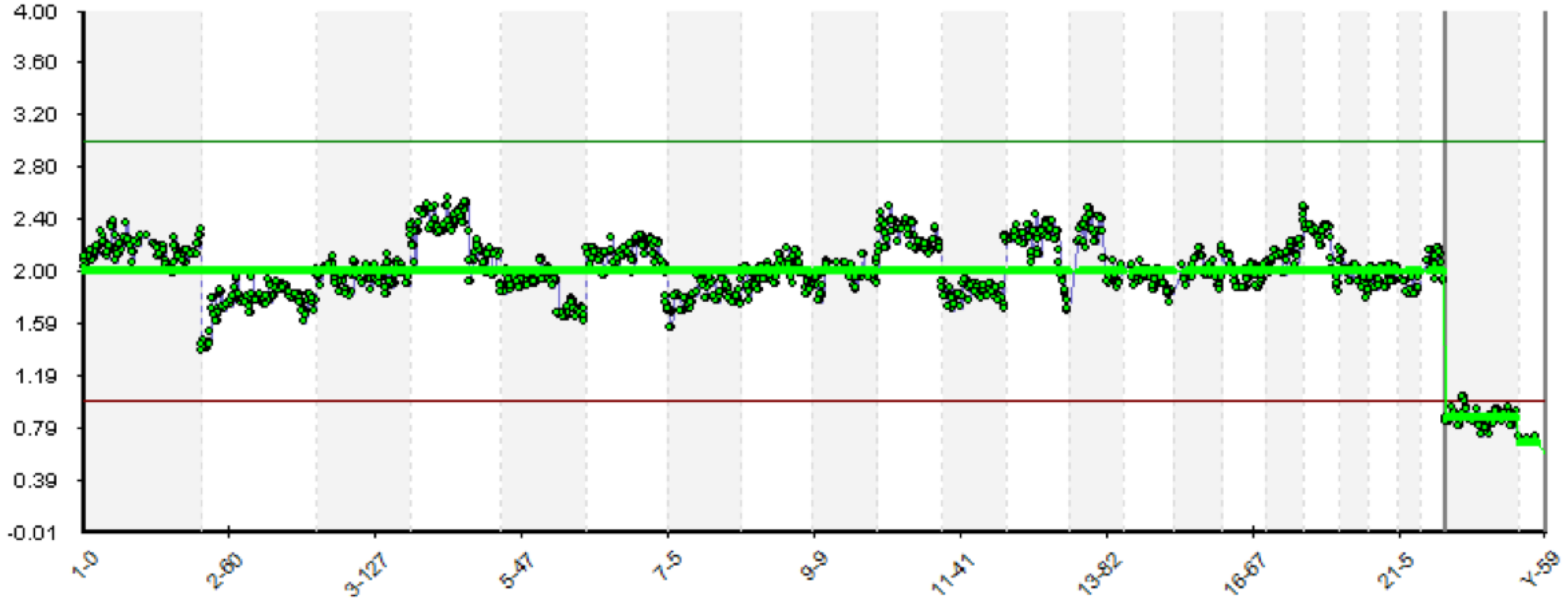

Embryo No13

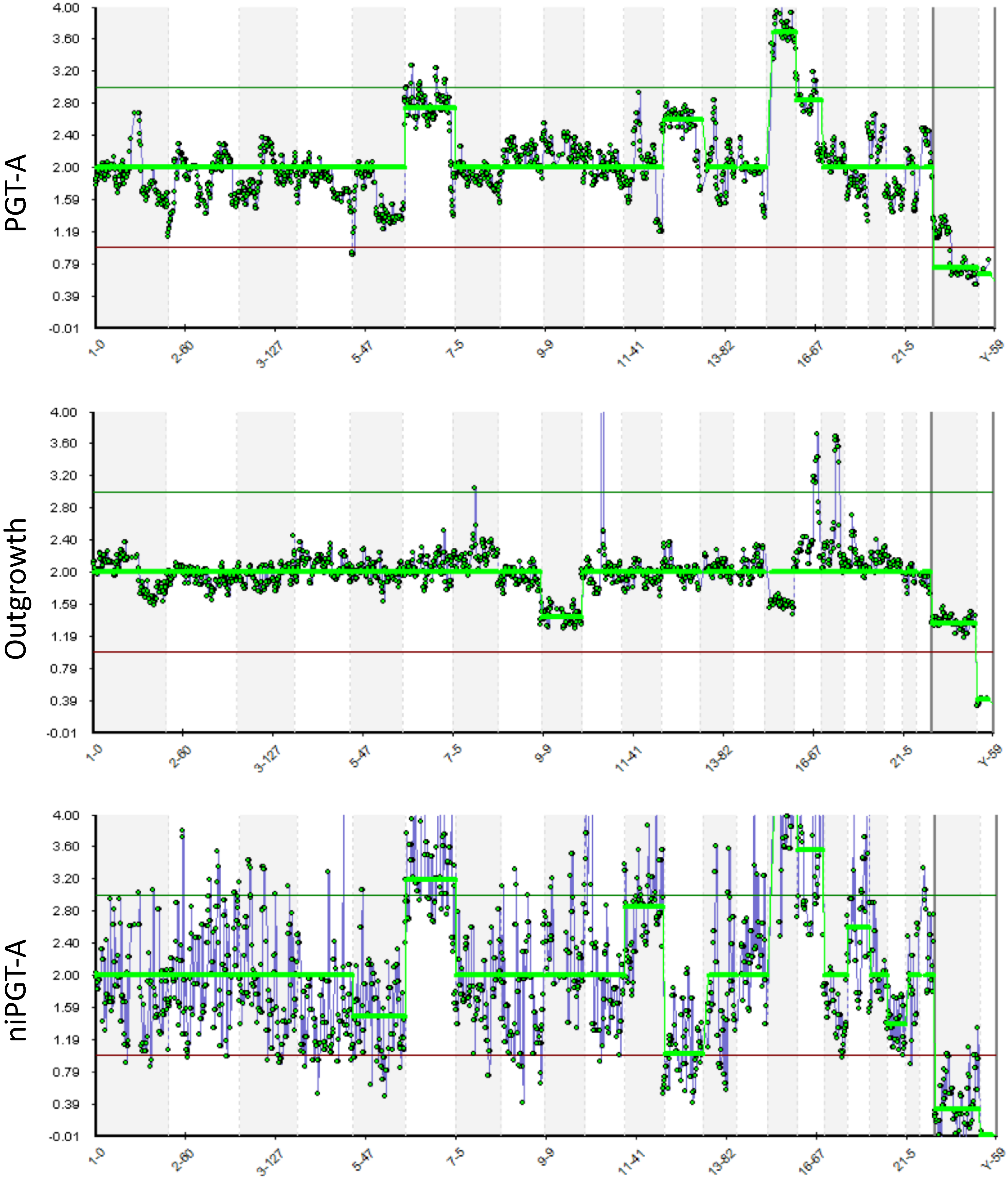
PGT-A
Outgrowth
niPGT-A
4.00
3.60
3.20
2.80
2.40
2.00
1.59
1.19
0.79
0.39
-0.01
1-0
2-60
3-127
5-47
7-5
9-9
11-41
13-82
16-67
21-5
Y-59

Embryo No14

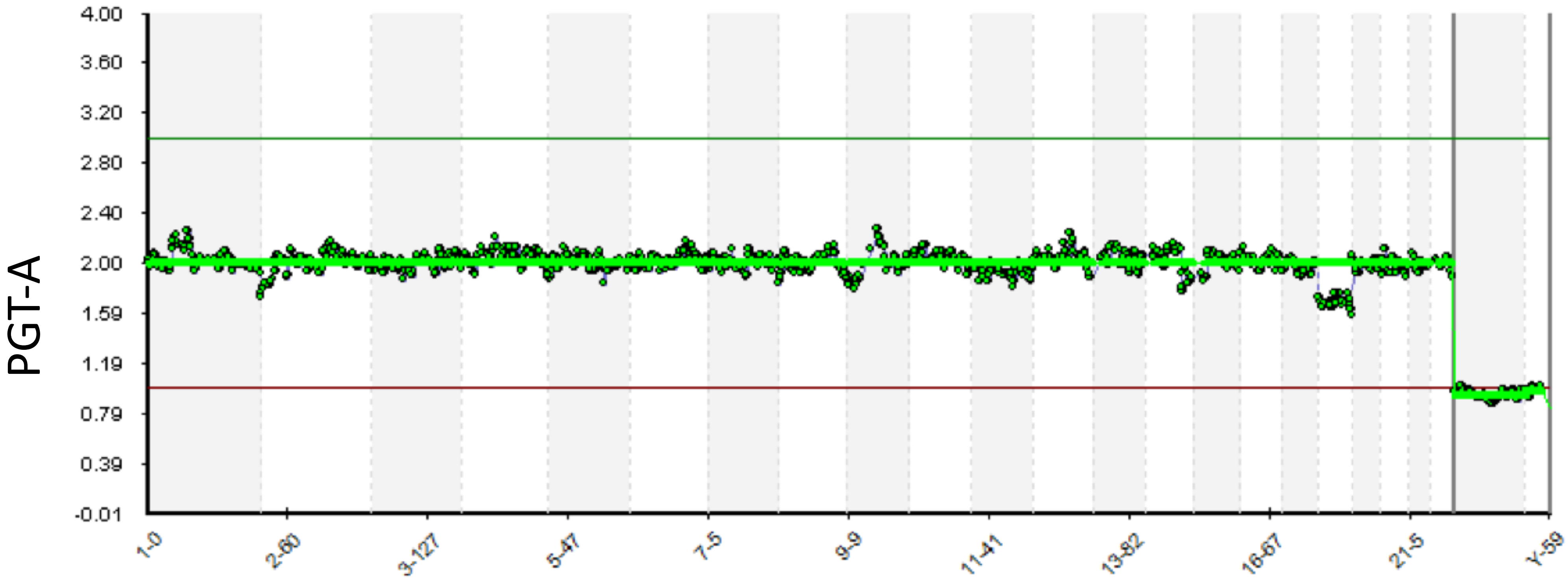

PGT-A
4.00
3.60
3.20
2.80
2.40
2.00
1.59
1.19
0.79
0.39
-0.01
1-0
2-60
3-127
5-47
7-5
9-9
11-41
13-82
16-67
21-5
Y-59

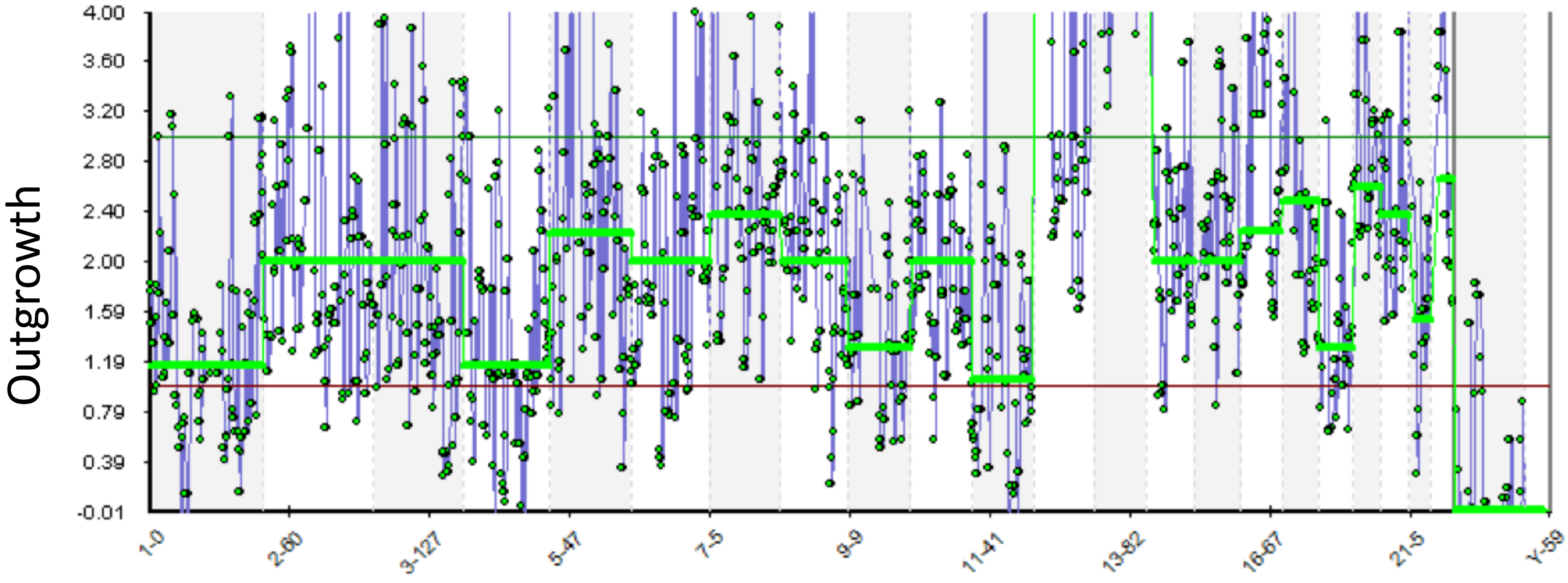

Outgrowth
4.00
3.60
3.20
2.80
2.40
2.00
1.59
1.19
0.79
0.39
-0.01
1-0
2-60
3-127
5-47
7-5
9-9
11-41
13-82
16-67
21-5
Y-59

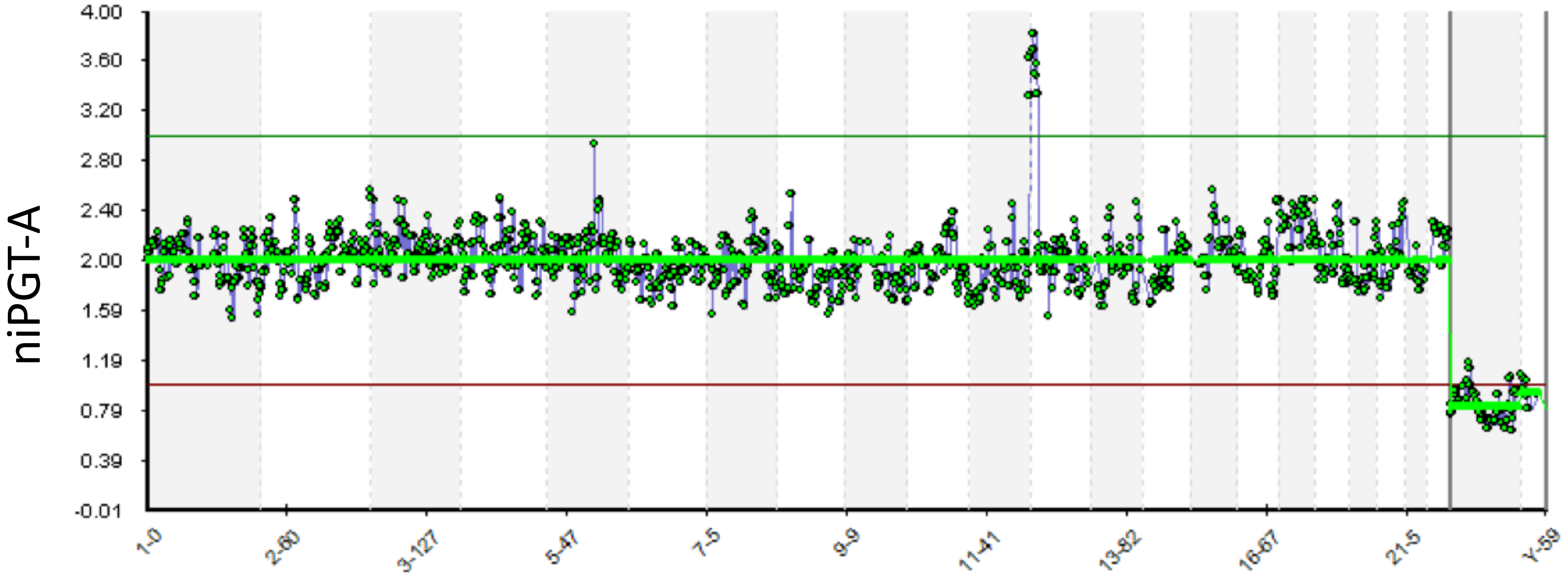

niPGT-A
4.00
3.60
3.20
2.80
2.40
2.00
1.59
1.19
0.79
0.39
-0.01
1-0
2-60
3-127
5-47
7-5
9-9
11-41
13-82
16-67
21-5
Y-59

Embryo No15

PGT-A

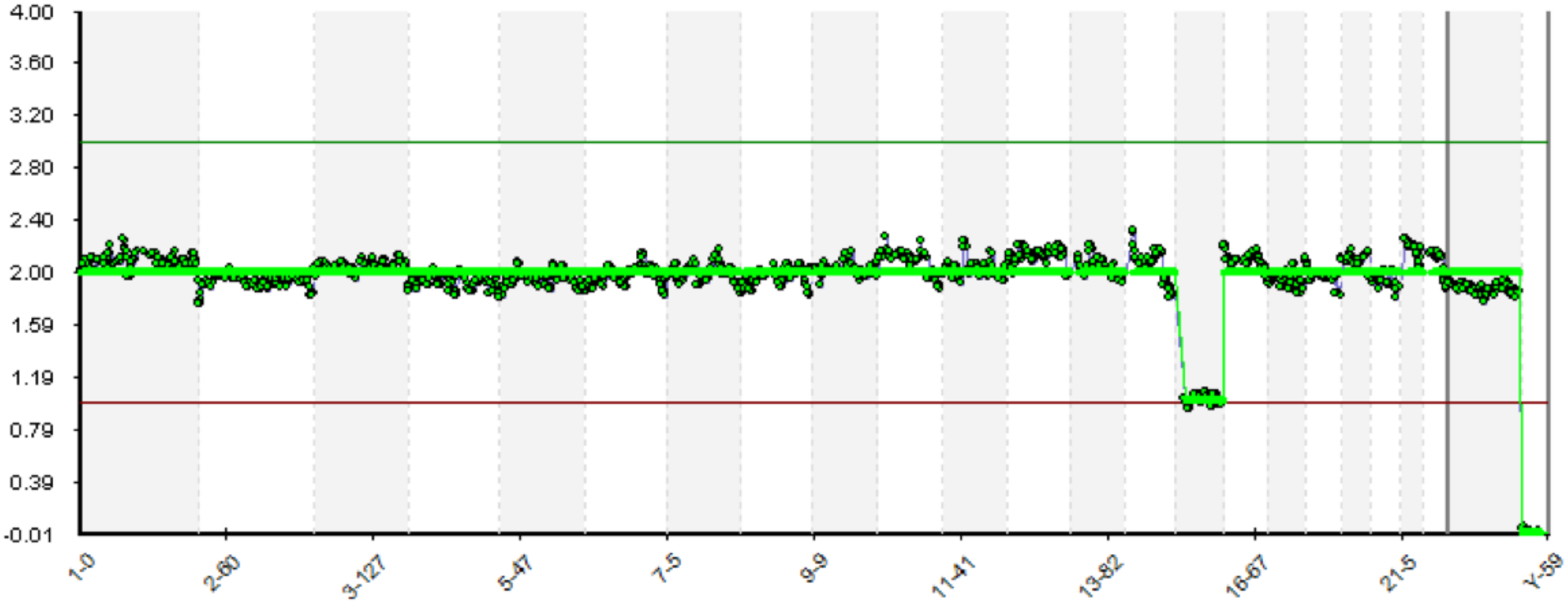

Outgrowth

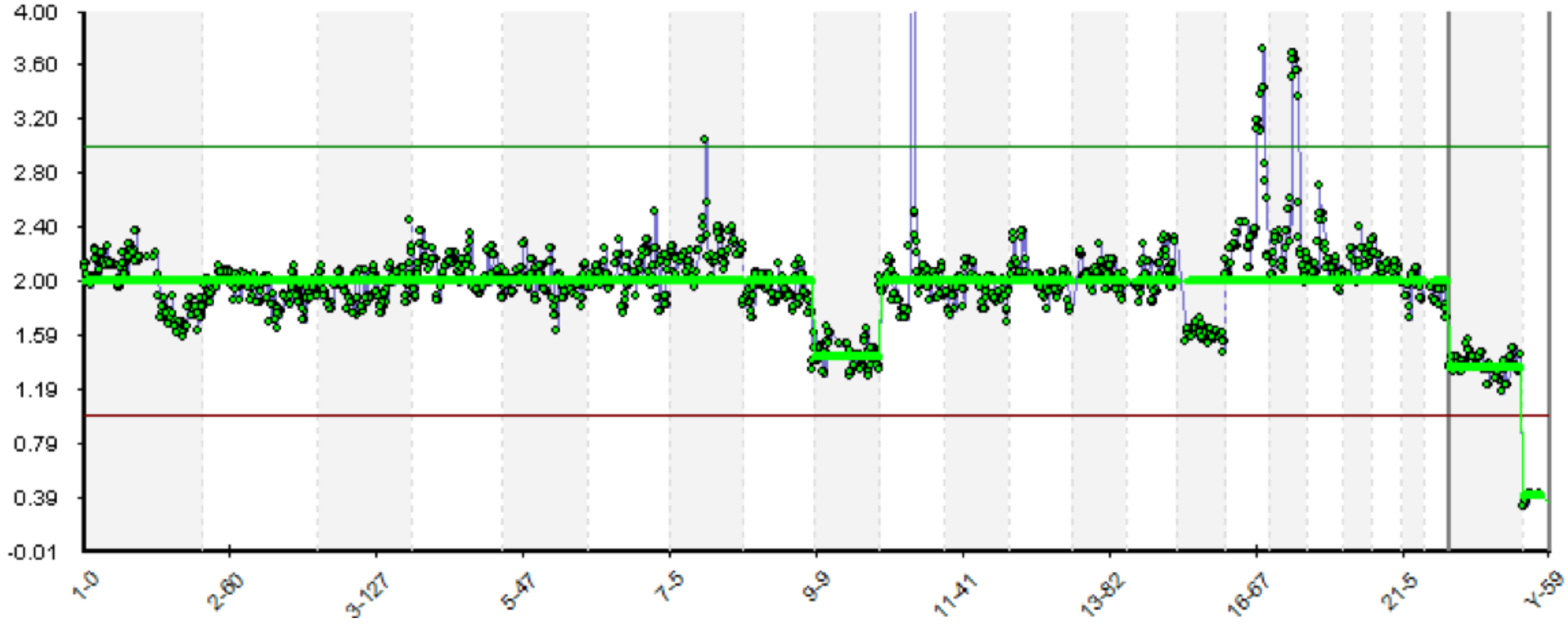

niPGT-A

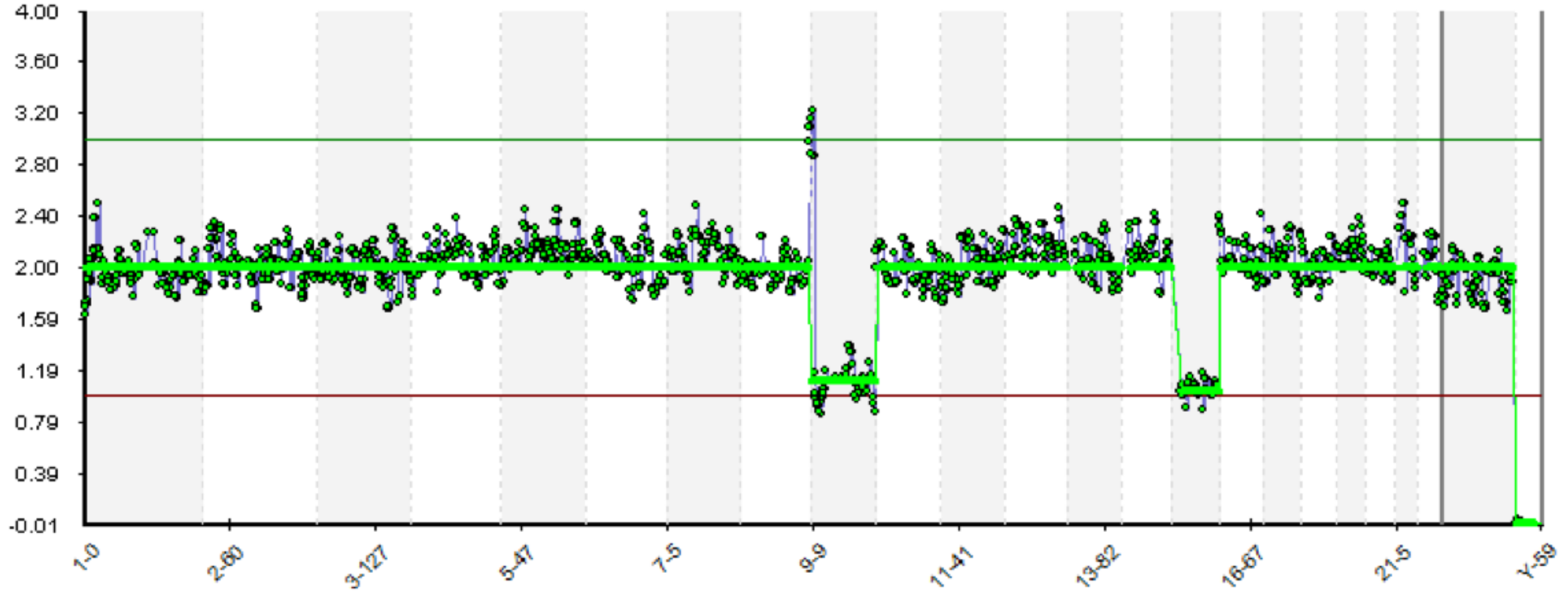

Embryo No16

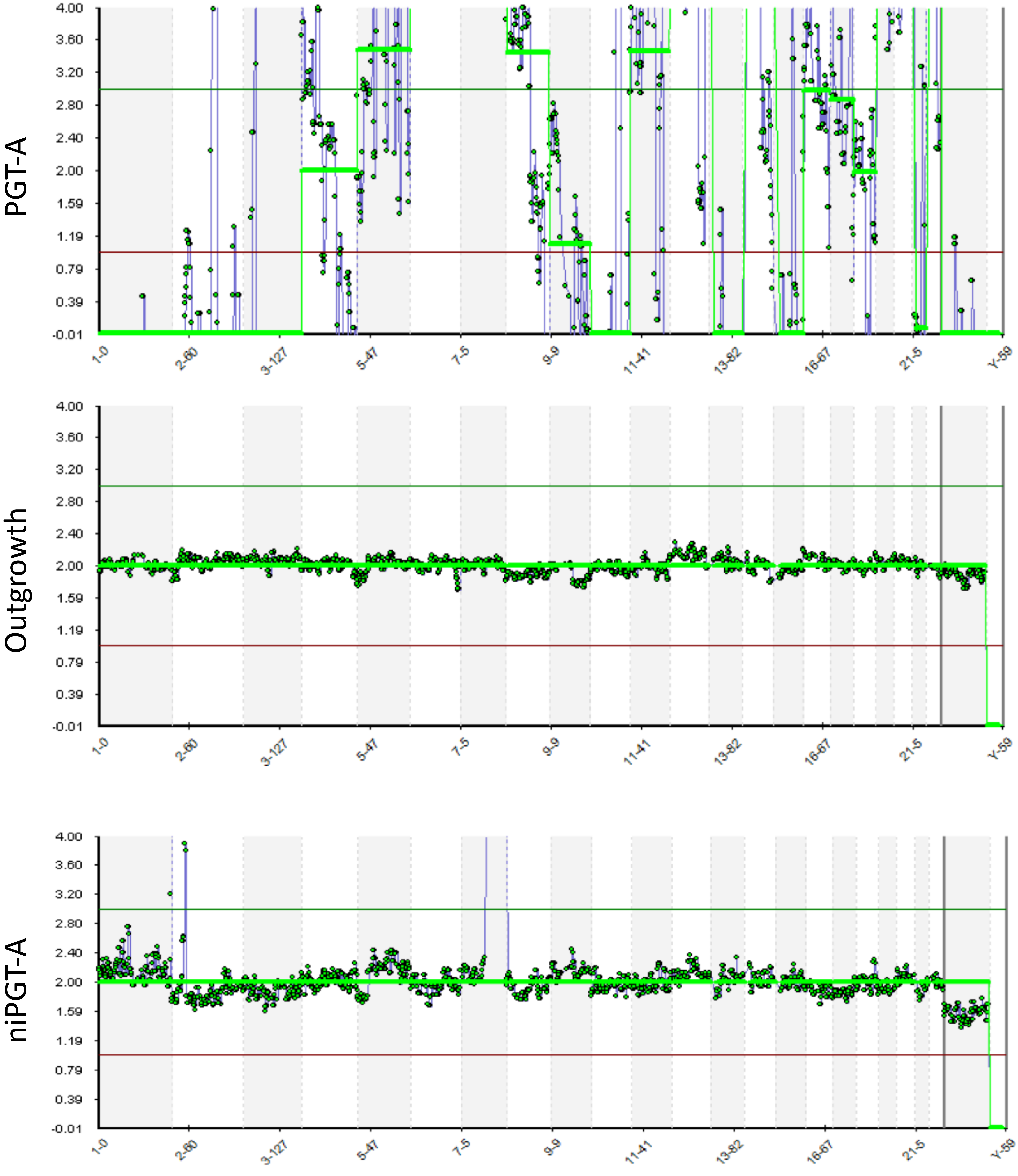
PGT-A
Outgrowth
niPGT-A
4.00
3.60
3.20
2.80
2.40
2.00
1.59
1.19
0.79
0.39
-0.01
1-0
2-60
3-127
5-47
7-5
9-9
11-41
13-82
16-67
21-5
Y-59

Embryo No17

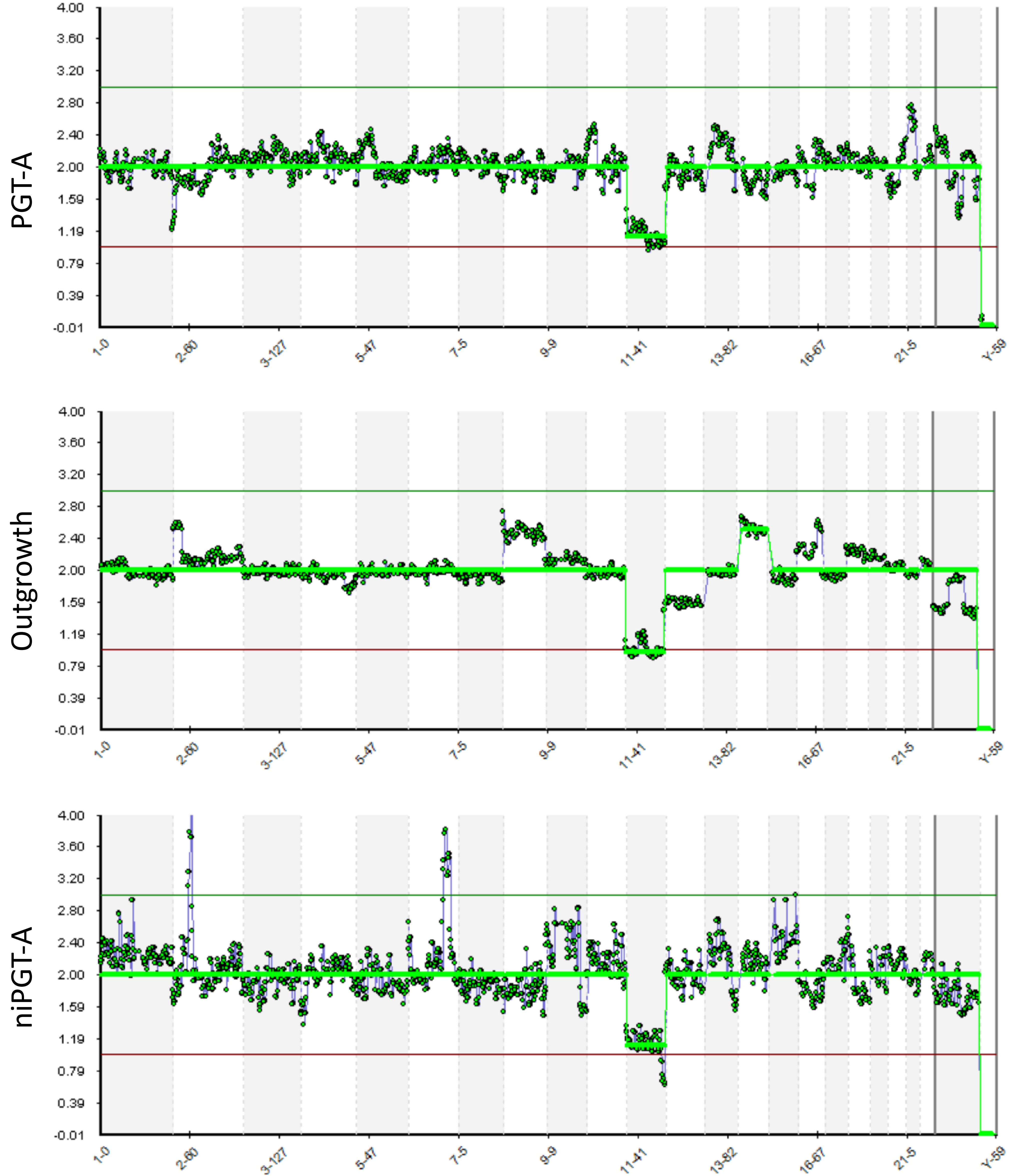
PGT-A
Outgrowth
niPGT-A
4.00
3.60
3.20
2.80
2.40
2.00
1.59
1.19
0.79
0.39
-0.01
1-0
2-60
3-127
5-47
7-5
9-9
11-41
13-82
16-67
21-5
Y-59

Embryo No18

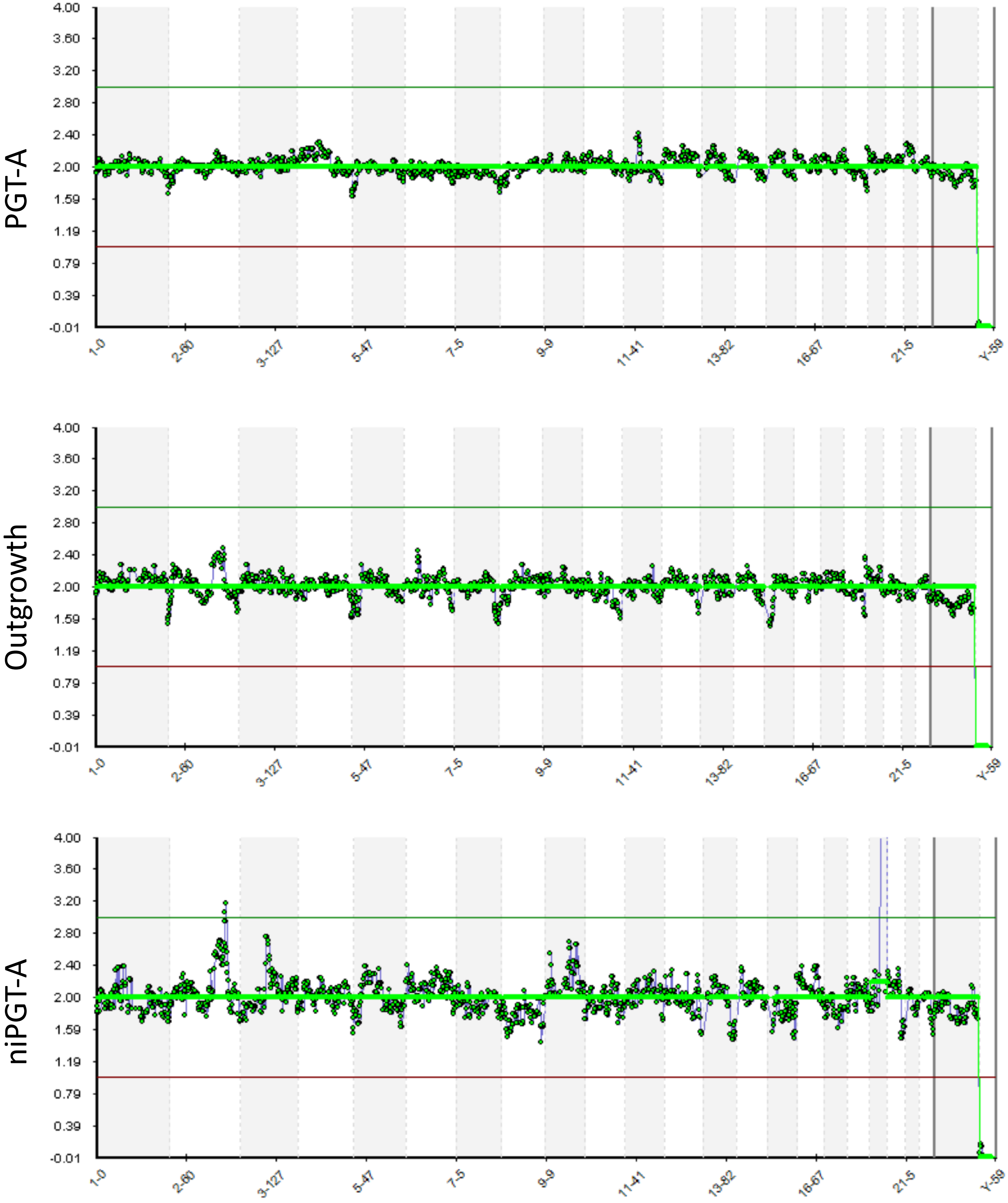

PGT-A
Outgrowth
niPGT-A
4.00
3.60
3.20
2.80
2.40
2.00
1.59
1.19
0.79
0.39
-0.01
1-0
2-60
3-127
5-47
7-5
9-9
11-41
13-82
16-67
21-5
Y-59

Embryo No19

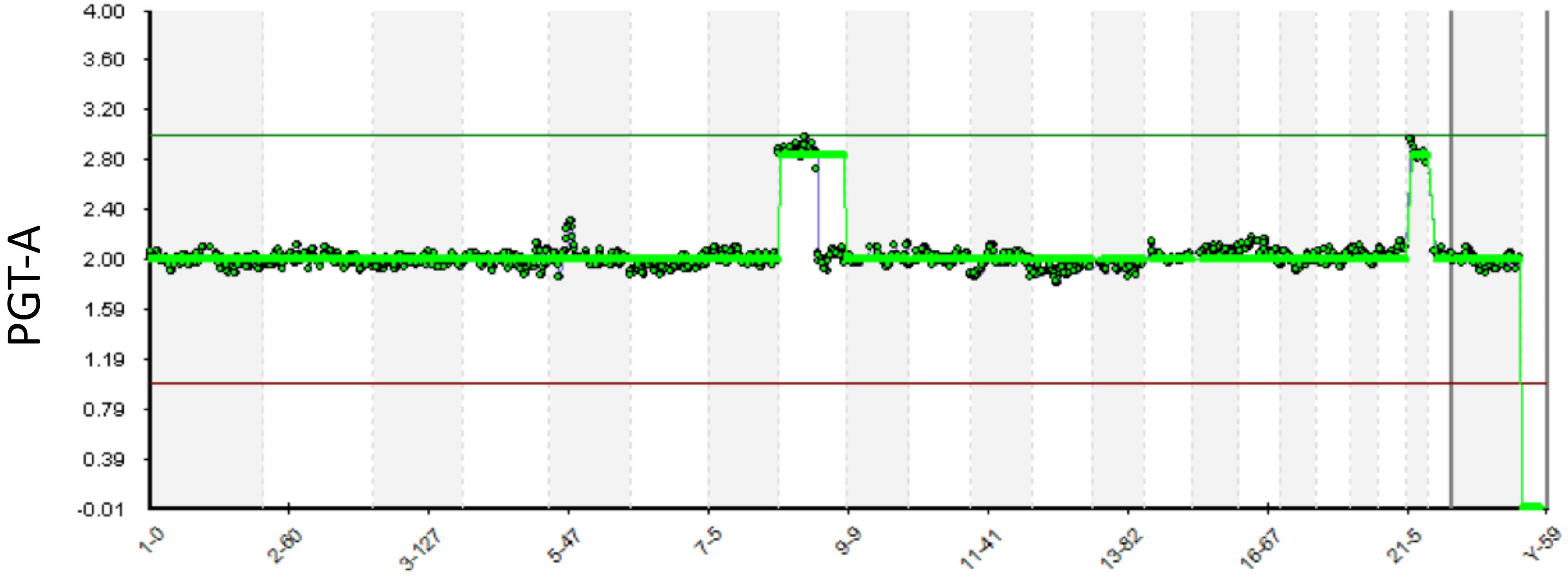
PGT-A
4.00
3.60
3.20
2.80
2.40
2.00
1.59
1.19
0.79
0.39
-0.01
1-0
2-60
3-127
5-47
7-5
9-9
11-41
13-82
16-67
21-5
Y-59

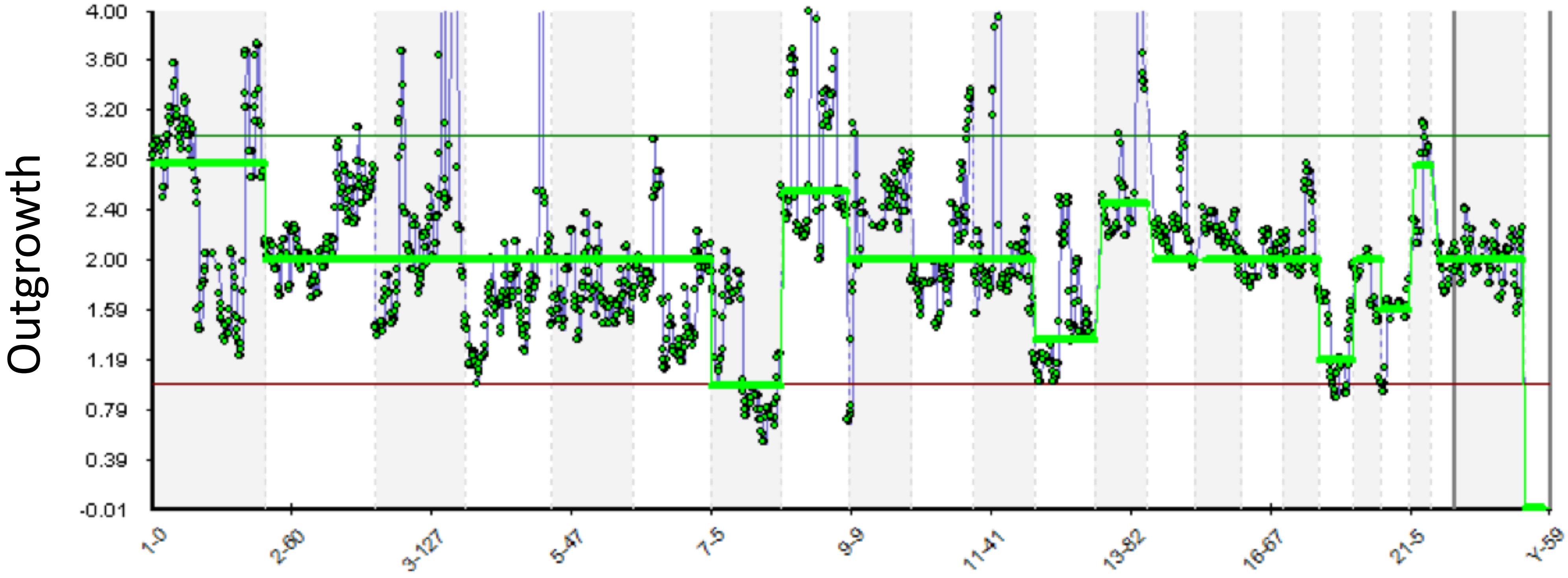
Outgrowth
4.00
3.60
3.20
2.80
2.40
2.00
1.59
1.19
0.79
0.39
-0.01
1-0
2-60
3-127
5-47
7-5
9-9
11-41
13-82
16-67
21-5
Y-59

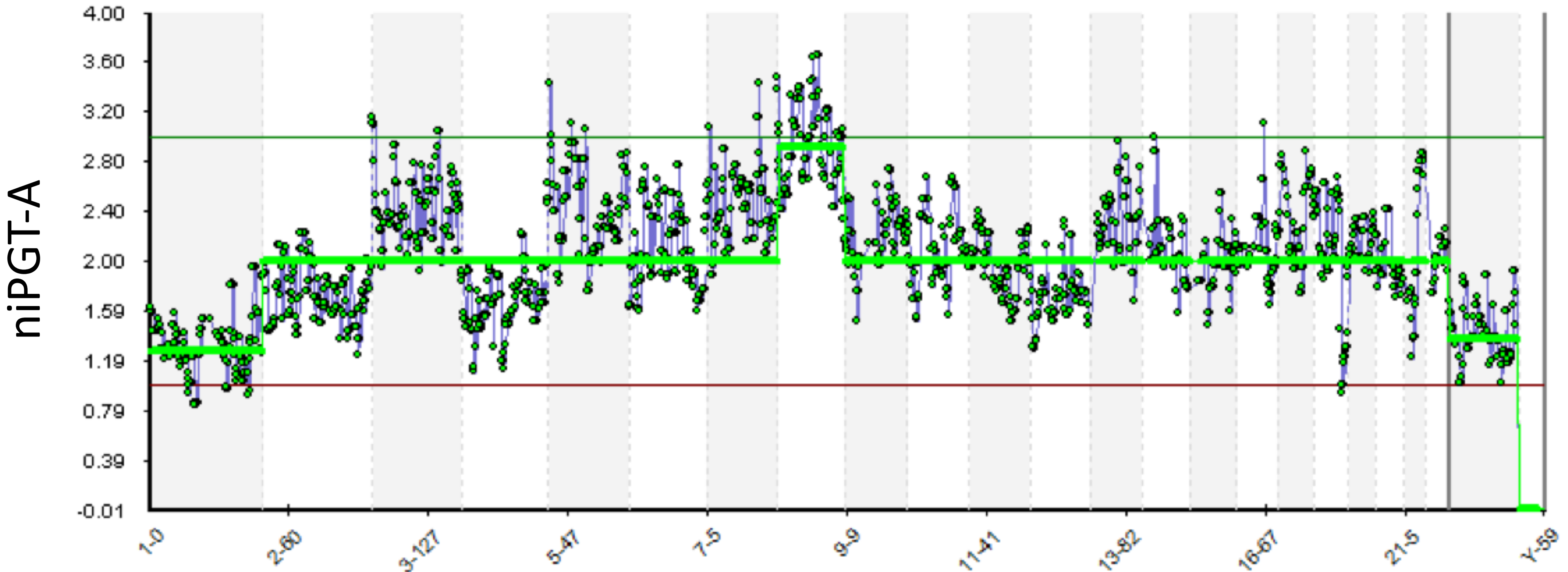
niPGT-A
4.00
3.60
3.20
2.80
2.40
2.00
1.59
1.19
0.79
0.39
-0.01
1-0
2-60
3-127
5-47
7-5
9-9
11-41
13-82
16-67
21-5
Y-59

Embryo No20

PGT-A

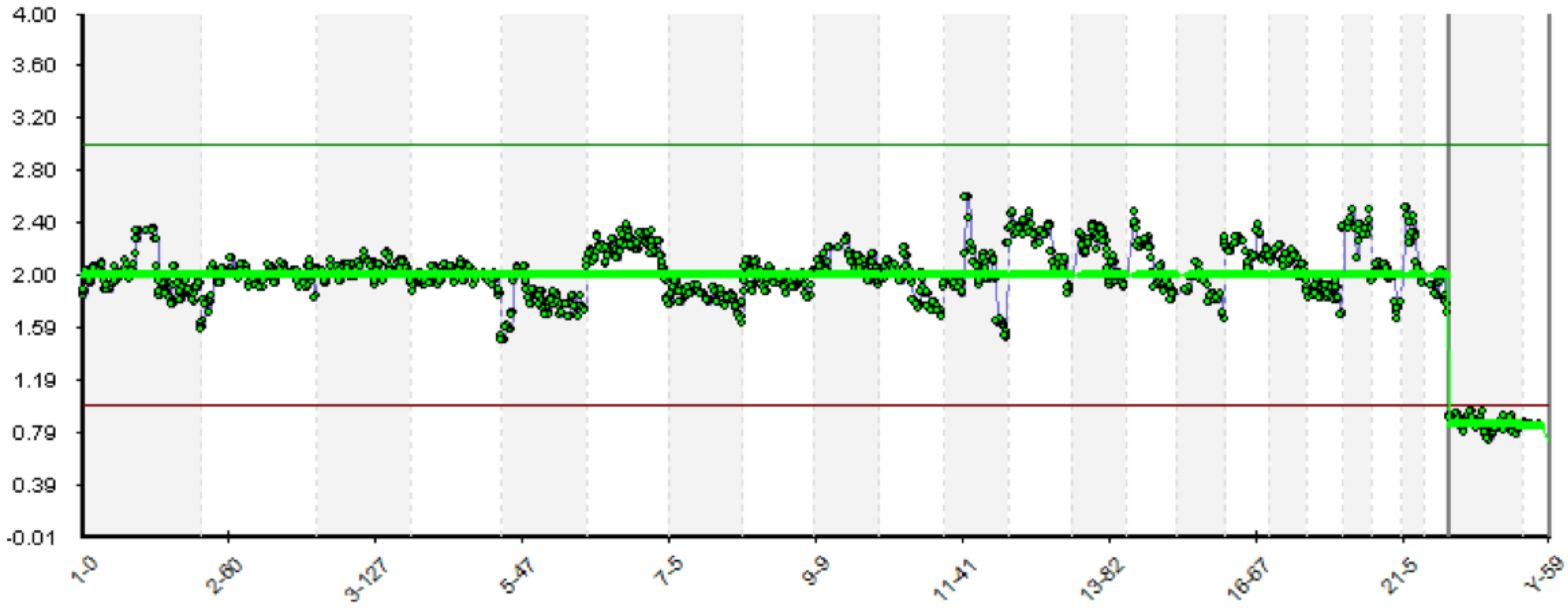

Outgrowth

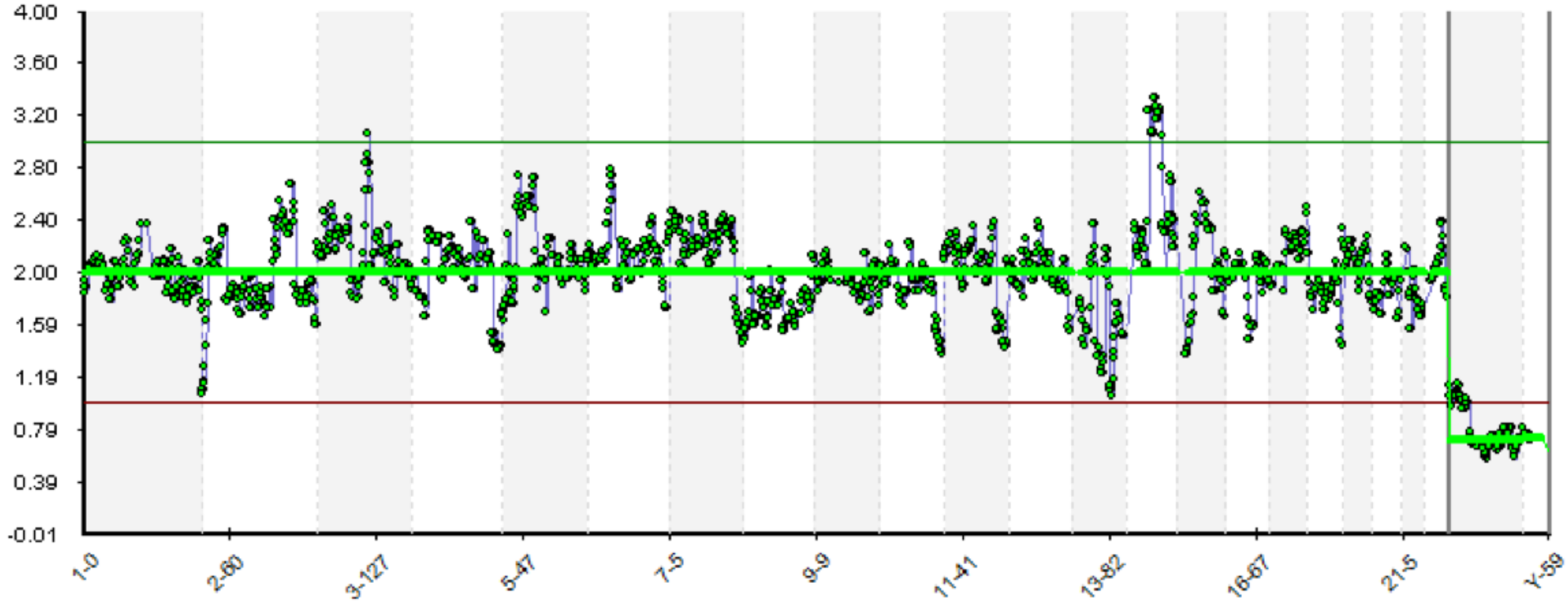

niPGT-A

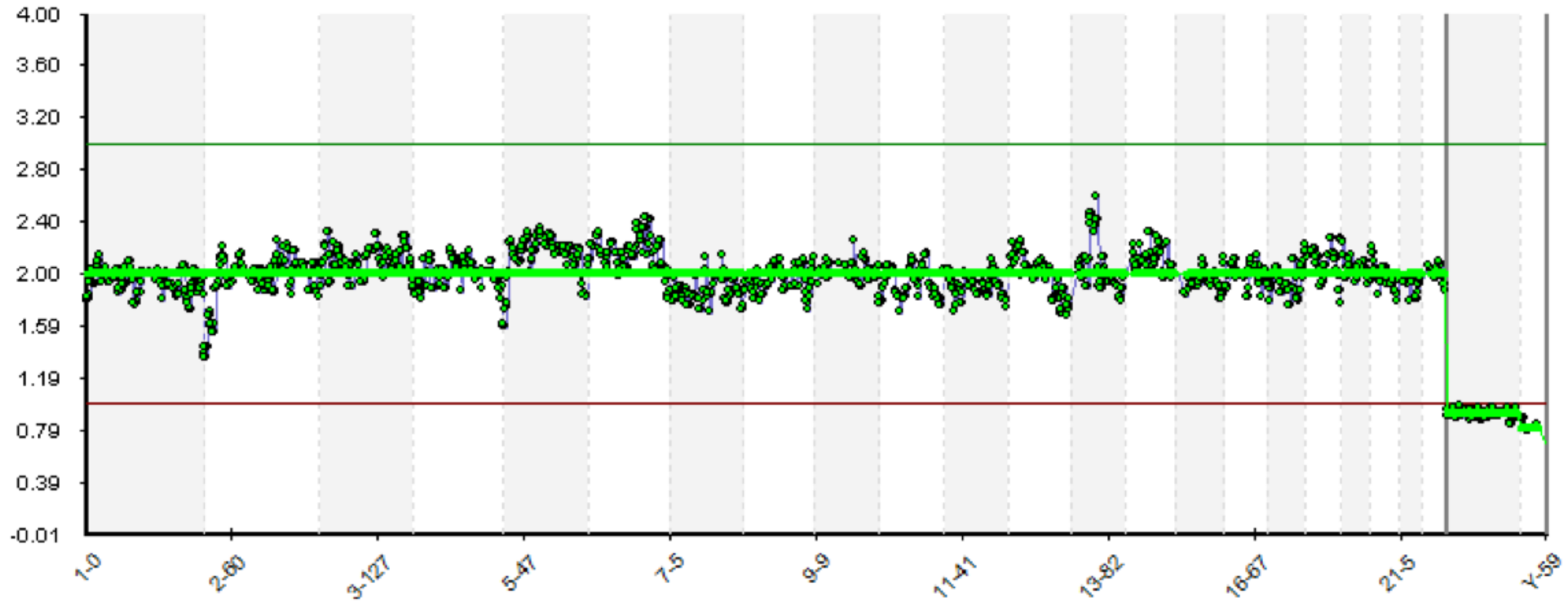

Supplement: S1 Fig — NGS was performed using a MiSeq testing device (Illumina, San Diego, CA, USA). The obtained data were analyzed using Bluefuse Multi software to obtain the karyotype information of the sample. https://doi.org/10.6084/m9.figshare.13488771. (PDF) [file pone.0246438.s001.pdf]
