# Supplementary material for: Cell-free DNA in spent culture medium effectively reflects the chromosomal status of embryos following culturing beyond implantation compared to trophectoderm biopsy
Source: PLoS One. 2021 Feb 11;16(2):e0246438. doi: 10.1371/journal.pone.0246438 (PMC7877764; doi:10.1371/journal.pone.0246438)
Supplement: S2 Table — Whole genome amplification (WGA) were performed using the SurePlex DNA Amplification System (Illumina, San Diego, CA, USA), according to the manufacturer’s recommended conditions. The DNA concentration of the product after amplification was measured using a Qubit 3.0 Fluorometer (ThermoFisher Scientific) with the Qubit dsDNA HS Assay kit (ThermoFisher Scientific). https://doi.org/10.6084/m9.figshare.13487427. (DOCX) [file pone.0246438.s003.docx]

**S2 Table. DNA concentration after WGA for PGT-A, Outgrowth, niPGT-A**

Whole genome amplification (WGA) were performed using the SurePlex DNA Amplification System (Illumina, San Diego, CA, USA), according to the manufacturer’s recommended conditions. The DNA concentration of the product after amplification was measured using a Qubit 3.0 Fluorometer (ThermoFisher Scientific) with the Qubit dsDNA HS Assay kit (ThermoFisher Scientific).

| **patient NO** | **embryo No** | **Time of  freezing(day)** | **PGT-A** | **outgrowth** | **niPGT-A** |
| --- | --- | --- | --- | --- | --- |
| 1 | *1* | 5 | 34 | 40.6 | 22 |
| 2 | *2* | 5 | 39 | 40.2 | 24 |
| 3 | *3* | 5 | 41 | 36.4 | 27.2 |
| 3 | *4* | 5 | 39.8 | 43.4 | 16.8 |
| 4 | *5* | 5 | 42.8 | 16.4 | 21.2 |
| 4 | *6* | 5 | 45.8 | 37.4 | 17.9 |
| 3 | *7* | 5 | 49.8 | 43.4 | 17.9 |
| 3 | *8* | 5 | 48 | 42.6 | 23.6 |
| 3 | *9* | 5 | 43.8 | 31.4 | 21.4 |
| 5 | *10* | 5 | 37.2 | 31 | 28.4 |
| 6 | *11* | 6 | 38 | 29 | 32.8 |
| 7 | *12* | 6 | 42.4 | 44.4 | 22 |
| 8 | *13* | 6 | 49.8 | 41.2 | 17.1 |
| 8 | *14* | 6 | 35.6 | 4.32 | 12.8 |
| 9 | *15* | 6 | 37 | 23.4 | 9.28 |
| 10 | *16* | 6 | 36.6 | 39.6 | 30.2 |
| 10 | *17* | 6 | 41 | 38.8 | 26 |
| 11 | *18* | 6 | 40.6 | 41 | 27.8 |
| 11 | *19* | 6 | 43.6 | 31.2 | 16.1 |
| 12 | *20* | 6 | 41.6 | 40.6 | 29.4 |
